# Targeting the mSWI/SNF complex in POU2F-POU2AF transcription factor-driven malignancies

**DOI:** 10.1016/j.ccell.2024.06.006

**Published:** 2024-07-18

**Authors:** Tongchen He, Lanbo Xiao, Yuanyuan Qiao, Olaf Klingbeil, Eleanor Young, Xiaoli S. Wu, Rahul Mannan, Somnath Mahapatra, Esther Redin, Hanbyul Cho, Yi Bao, Malathi Kandarpa, Jean Ching-Yi Tien, Xiaoju Wang, Sanjana Eyunni, Yang Zheng, NamHoon Kim, Heng Zheng, Siyu Hou, Fengyun Su, Stephanie J. Miner, Rohit Mehra, Xuhong Cao, Chandrasekhar Abbineni, Susanta Samajdar, Murali Ramachandra, Saravana M. Dhanasekaran, Moshe Talpaz, Abhijit Parolia, Charles M. Rudin, Christopher R. Vakoc, Arul M. Chinnaiyan

**Affiliations:** 1 Michigan Center for Translational Pathology, University of Michigan, Ann Arbor, MI 48109, USA; 2 Department of Pathology, University of Michigan, Ann Arbor, MI 48109, USA; 3 Department of Urology, Xiangya Hospital, Central South University, Changsha, Hunan 410008, China; 4 Rogel Cancer Center, University of Michigan, Ann Arbor, MI 48109, USA; 5 Cold Spring Harbor Laboratory, Cold Spring Harbor, NY 11724, USA; 6 Department of Medicine, Memorial Sloan Kettering Cancer Center, New York, NY 10065, USA; 7 Department of Internal Medicine, Division of Hematology and Oncology, University of Michigan, Ann Arbor, MI 48109, USA; 8 Department of Biostatistics, School of Public Health, University of Michigan, Ann Arbor, MI 48109, USA; 9 Howard Hughes Medical Institute, University of Michigan, Ann Arbor, MI 48109, USA; 10 Aurigene Oncology Limited, Bangalore 560100, India; 11 Department of Urology, University of Michigan, Ann Arbor, MI 48109, USA; 12 Weill Cornell Medicine Graduate School of Medicine Sciences, New York, NY 10065, USA; 13 These authors contributed equally; 14 Lead contact

## Abstract

The POU2F3-POU2AF2/3 transcription factor complex is the master regulator of the tuft cell lineage and tuft cell-like small cell lung cancer (SCLC). Here, we identify a specific dependence of the POU2F3 molecular subtype of SCLC (SCLC-P) on the activity of the mammalian switch/sucrose non-fermentable (mSWI/SNF) chromatin remodeling complex. Treatment of SCLC-P cells with a proteolysis targeting chimera (PROTAC) degrader of mSWI/SNF ATPases evicts POU2F3 and its coactivators from chromatin and attenuates downstream signaling. B cell malignancies which are dependent on the POU2F1/2 cofactor, POU2AF1, are also sensitive to mSWI/SNF ATPase degraders, with treatment leading to chromatin eviction of POU2AF1 and IRF4 and decreased IRF4 signaling in multiple myeloma cells. An orally bioavailable mSWI/SNF ATPase degrader significantly inhibits tumor growth in preclinical models of SCLC-P and multiple myeloma without signs of toxicity. This study suggests that POU2F-POU2AF-driven malignancies have an intrinsic dependence on the mSWI/SNF complex, representing a therapeutic vulnerability.

## In brief

He et al. reveal a promising avenue of treatment for small cell lung cancer and multiple myeloma driven by POU2F/POU2AF. The study highlights the potential of targeting the mSWI/SNF complex to impede oncogenic POU2F/POU2AF signaling and tumor growth, offering hope for improved therapies in these aggressive cancer types.

## Graphical Abstract

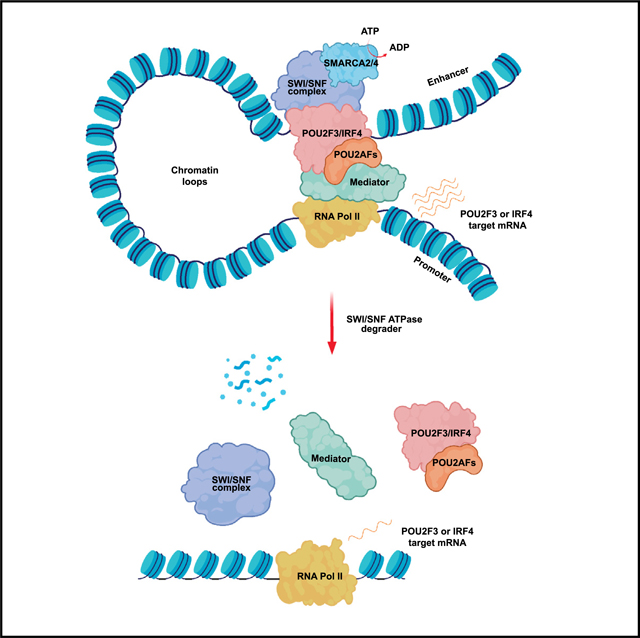

## INTRODUCTION

Small cell lung cancer (SCLC) is an aggressive, fast-evolving subtype of lung cancer with a high growth rate and early metastasis propensity, often resulting in a more advanced disease stage at diagnosis.^1,2^ Consequently, the overall prognosis for SCLC is generally poorer compared to non-small cell lung cancer (NSCLC).^3^ Unlike NSCLC, where substantial progress has been achieved with immune checkpoint blockade therapies, effective targeted therapies for SCLC remain elusive.^4^ Comprehensive genome sequencing of SCLC tumors has revealed a high mutational load in this disease, with most tumors possessing inactivating mutations or deletions of *RB1* and *TP53*, but few actionable targets have been identified.^5^ Thus, there is an urgent need for innovative therapeutic strategies that address the distinct biology of SCLC and enhance patient outcomes.

Prior analysis of human SCLC tumors reveals that SCLC can be characterized by the expression pattern of certain transcription factors (TFs) or transcriptional regulators, including ASCL1 (achaete-scute family bHLH transcription factor 1), NeuroD1 (neurogenic differentiation factor 1), POU2F3 (POU domain class 2 transcription factor 3; also known as OCT-11), and YAP1 (yes-associated protein 1), exemplifying SCLC as a TF-driven malignancy.^6–9^ ASCL1-driven SCLC (SCLC-A) and NeuroD1-driven SCLC (SCLC-N) manifest a neuroendocrine phenotype, while POU2F3-driven SCLC (SCLC-P) is characterized as a tuft cell-like variant.^9^ Prior studies reveal that POU domain class 2 TFs uniquely rely on coactivators to achieve their lineage-defining functions in B cells.^10–13^ More recently, in tuft cell-like SCLC cells, the coactivators of POU2F3 (POU2AF2 and POU2AF3) were found to endow POU2F3 with a critical transactivation domain by forming a master regulator complex, which supports enhancer-mediated cancer-promoting gene activation in SCLC-P cells.^14–16^ This indicates a potential therapeutic vulnerability in patients with tuft cell-like SCLC whereby strategies aimed at blocking POU2F3-POU2AF2/3 function may lead to clinical benefit.

The mammalian switch/sucrose non-fermentable (mSWI/SNF) chromatin remodeling complex acts as a pivotal regulator of gene expression and chromatin architecture, thereby orchestrating fundamental cellular processes crucial for homeostasis and development.^17^ The ATPase subunit of this complex harnesses energy from ATP hydrolysis to reposition or eject nucleosomes at non-coding regulatory elements, facilitating unobstructed DNA access for the transcriptional machinery.^18–20^ Recent investigations have elucidated alterations in the genes encoding constituent subunits of the mSWI/SNF complex in over 25% of human malignancies.^21,22^ Our group recently discovered that androgen receptor (AR)-driven prostate cancer cells are preferentially dependent on the chromatin remodeling function of the mSWI/SNF complex.^23^ We identified a mSWI/SNF ATPase proteolysis targeting chimera (PROTAC) degrader that dislodges AR and its cofactors from chromatin, disabling their core enhancer circuitry, and attenuating downstream oncogenic gene programs.^23^ Similar observations have been reported in other TF-driven malignancies like acute myeloid leukemia,^24,25^ highlighting the broad applicability of targeting the mSWI/SNF complex in a variety of malignancies.

In this study, we identified an enhanced dependency on the mSWI/SNF complex in POU2F3-driven SCLC cells through CRISPR screening and pharmacological validation. Epigenomics analyses revealed that inactivation of the mSWI/SNF complex preferentially obstructed chromatin accessibility of POU2F3 complexes, leading to a dramatic downregulation of POU2F3 signaling. Critically, treatment with an orally bioavailable mSWI/SNF ATPase PROTAC degrader resulted in significant tumor growth inhibition in preclinical models of POU2F3-driven SCLC without significant effects in other subtypes of SCLC xenografts. Furthermore, our investigations extended to other POU2AF1 complex-dependent B cell malignancies, mainly multiple myeloma, wherein sensitivity to the mSWI/SNF ATPase PROTAC degrader was observed *in vitro* and *in vivo*. These findings collectively show the potential of targeting the mSWI/SNF complex in POU2F-POU2AF-driven malignancies and suggest that development of mSWI/SNF degraders should be pursued as targeted therapies for patients with these types of cancers.

## RESULTS

### Dependence of SCLC-P cells on the mSWI/SNF complex

**Figure 1.**
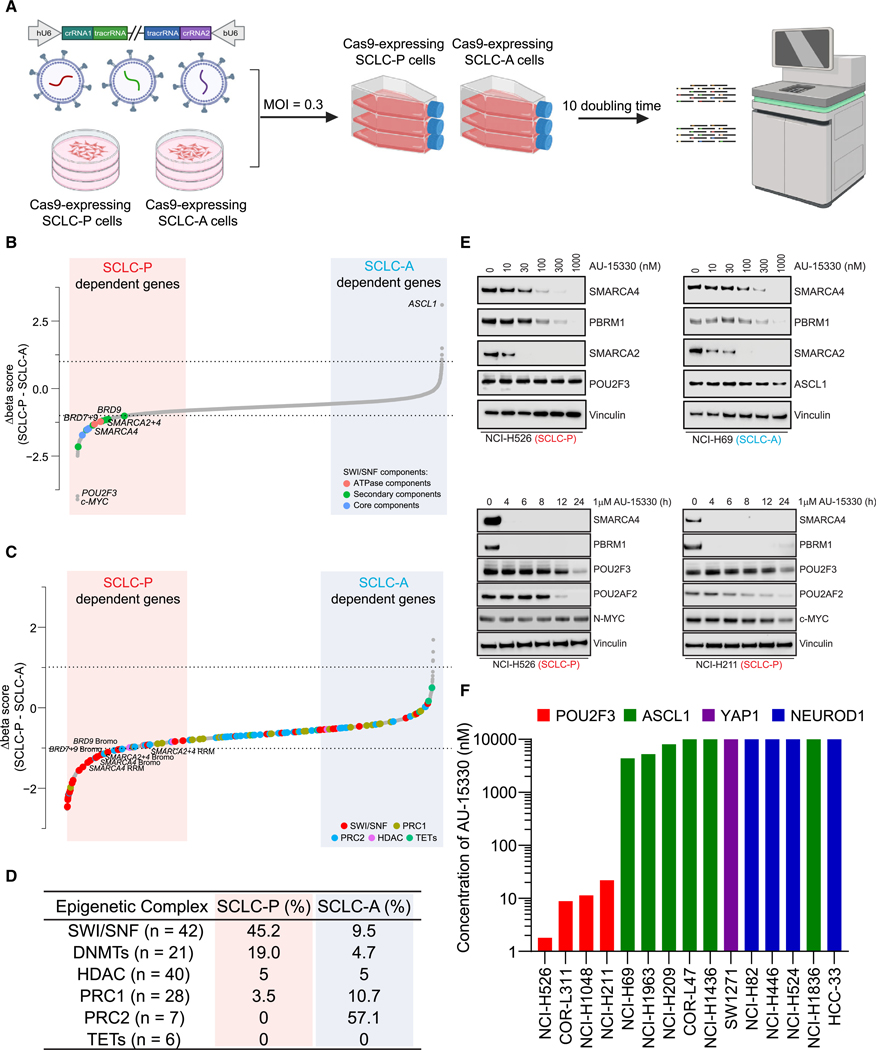
Dependence of SCLC-P cells on the mSWI/SNF complex (A) A schematic representation of the dual-sgRNA, domain-focused CRISPR screening designed to identify druggable epigenetic targets selective for SCLC subtypes. (B) Beta scores pertaining to all CRISPR screen targeted genes across both SCLC-P and SCLC-A cell lines (*n* = 5,308). (C) Beta scores highlighting epigenetic regulators in SCLC-P and SCLC-A cell lines (*n* = 3292). (D) Percentage of different epigenetic complexes in SCLC-P and SCLC-A cell lines (top 10% for each). PRC1, polycomb repressive complex 1; PRC2, polycomb repressive complex 2; HDAC, histone deacetylase; TET, ten-eleven translocation family proteins. (E) Immunoblot analysis of indicated proteins in SCLC-P and SCLC-A cells post-treatment with varying time points or concentrations of AU-15330. Vinculin serves as the control for protein loading in all immunoblots. (F) Compilation of the IC_50_ values for AU-15330 in SCLC cell lines representing four molecular subtypes. See also Figure S1 and Table S1.

SCLCs are genetically driven by loss of function (LOF) alterations in tumor suppressor genes *RB1* and *TP53*,^5^ with distinct expression patterns of certain TFs or transcriptional regulators leading to four molecular subtypes (SCLC-A, SCLC-N, SCLC-P, and SCLC-Y (YAP1)).^6^ Functional genomics analyses have underscored the critical roles of these TFs or coactivators in each SCLC molecular subtype. However, unlike kinases, many TFs have been perceived as undruggable targets due to their enrichment of intrinsically disordered regions within their structures, indicating potential challenges in devising ASCL1 or POU2F3-direct targeting strategies. Considering this, we hypothesized that druggable targets selective to SCLC subtypes could be identified via a loss-of-function CRISPR-Cas9 screen. Accordingly, we conducted a functional domain-targeted CRISPR-Cas9 screen co-targeting paralog pairs of kinases, phosphatases, epigenetic regulators, and DNA binding proteins in three SCLC-A and three SCLC-P cell lines (Figure 1A). Dependency scores (beta scores) for 4,341 single-gene and 4,387 double-gene knockouts were calculated using MAGeCK.^26^ Comparing beta scores between SCLC-A and SCLC-P cell lines, we observed dramatic dependency differences for lineage TFs ASCL-1 and POU2F3. Surprisingly, we also identified a strong dependency bias of multiple components of the mSWI/SNF complex in SCLC-P cells (Figures 1B–1D, S1A, and S1B, Table S1).

We hypothesized that this selective dependency might originate from a POU2F3-imposed requirement on the mSWI/SNF complex. Among the mSWI/SNF complex components, only ATPases and bromodomain containing 9 (BRD9) were found to be directly targetable by recently developed PROTAC degraders, which have been engineered to induce target protein degradation through the ubiquitin-proteasome system (Figure S1B, Table S1).^27,28^ Our team recently showcased the promising anti-tumor efficacy of the PROTAC degrader targeting the mSWI/SNF ATPase subunit in preclinical models of AR-driven prostate cancer.^23^ Here, we evaluated the efficacy of this mSWI/SNF ATPase PROTAC degrader, AU-15330, across a spectrum of SCLC cell lines. AU-15330 treatment resulted in time and dose-dependent degradation of mSWI/SNF ATPases (SMARCA2 and SMARCA4) and PBRM1 in cell lines encompassing different molecular subtypes of SCLC (Figures 1E and S1C). Protein levels of POU2F3 and its coactivator POU2AF2 were also decreased in SCLC-P cells treated with AU-15330 at extended time points (12 and 24 h, Figures 1E and S1C). Despite degradation of target mSWI/SNF ATPase proteins across subtypes, AU-15330 exhibited a preferential growth inhibitory effect and induced apoptosis in SCLC-P cells compared to all non-POU2F3 SCLC cell line models (Figures 1F and S1D–S1F). Furthermore, analysis of publicly available SCLC patient data showed that SCLC-A patients had a higher frequency of mutations in mSWI/SNF components compared to SCLC-P patients (Figure S1G). Taken together, our functional CRISPR-Cas9 screen, complemented by secondary pharmacological validation, pinpointed the mSWI/SNF complex and its catalytic ATPase subunit as epigenetic dependencies in SCLC-P cells.

### Mechanism of action of mSWI/SNF complex inactivation in SCLC-P cells

**Figure 2.**
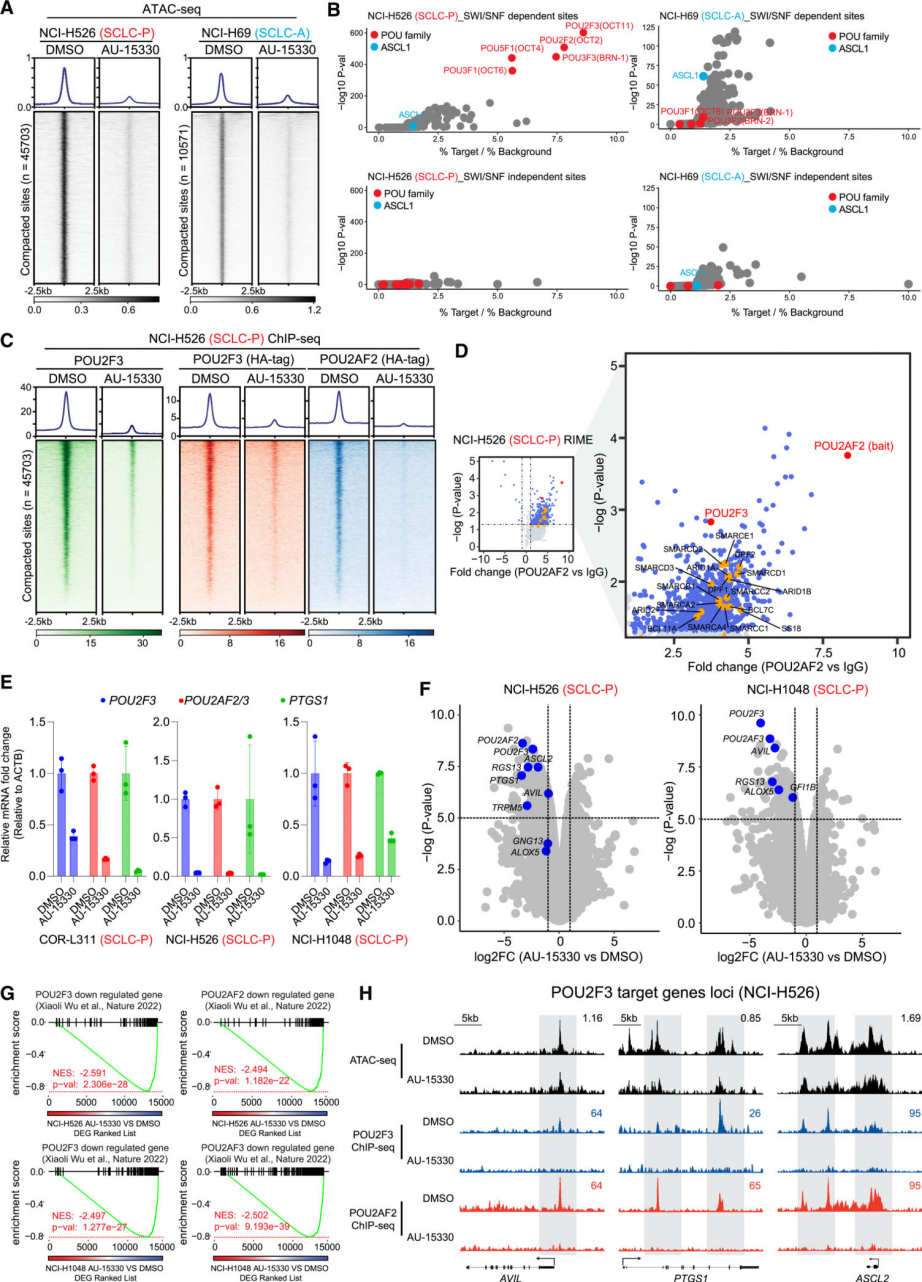
The POU2F3 transcription factor complex is evicted from chromatin in SCLC-P cells upon mSWI/SNF ATPase degradation (A) Visualization of ATAC-seq read-density in NCI-H526 (SCLC-P) and NCI-H69 (SCLC-A) cells post-treatment for 4 h with either vehicle or 1 μM AU-15330 (*n* =2 biological replicates). (B) Analysis of fold change and significance level for HOMER motifs that are enriched within sites dependent and independent of the mSWI/SNF complex in NCI-H526 and NCI-H69 cells. (C) ChIP-seq read-density heatmaps representing POU2F3 (green), HA-POU2F3 (red), and HA-POU2AF2 (blue) at AU-15330-loss genomic sites in NCI-H526 cells following treatment with DMSO or AU-15330. (D) Volcano plot detailing proteins that interact with POU2AF2, as identified by POU2AF2 RIME analysis in NCI-H526 cells. mSWI/SNF components highlighted in orange (*n* = 3 biological replicates). (E) Expression levels of *POU2F3*, *POU2AF2/3*, and *PTGS1* as assessed by QPCR (normalized to *ACTB*) in the indicated cell lines after being treated for 12 h with vehicle or 1 μM AU-15330. Data are presented as mean ± SD (*n* = 3 biological replicates). (F) Volcano plot visualizing the overall transcriptomic alterations as assessed by RNA-seq in NCI-H526 and NCI-H1048 cells post-treatment for 12 h with vehicle or 1 μM AU-15330. Canonical POU2F3 target genes are highlighted in blue (*n* = 2 biological replicates). (G) GSEA plots illustrating genes regulated by POU2F3 and its coactivators POU2AF2 and POU2AF3. The plots employ a gene signature ranked by fold change in AU-15330-treated NCI-H526 and NCI-1048 cells. DEG, differentially expressed gene. (H) Combined ATAC-seq and ChIP-seq tracks for *AVIL*, *PTGS1*, and *ASCL2* in NCI-H526 with and without AU-15330 treatment. See also Figures S1–S3 and Table S1.

Experiments were next performed to elucidate the mechanism of action underlying the selective growth inhibitory effects of the mSWI/SNF ATPase PROTAC degrader in SCLC-P cells. Given the primary role of the mSWI/SNF complex in modulating chromatin accessibility by altering nucleosome positioning along DNA, we employed assay for transposase-accessible chromatin using sequencing (ATAC-seq) in SCLC-P and SCLC-A cells post AU-15330 treatment. As depicted in Figures 2A and S1H, 4 h treatment with AU-15330 triggered rapid and genome-wide chromatin accessibility loss at regulatory regions in both SCLC-P and SCLC-A cells. *De novo* motif analysis of the sites affected by AU-15330 revealed that POU motif-containing sites were predominantly affected across the genome in SCLC-P cells (Figures 2B, S1I, and S2A–S2D). Conversely, the ASCL1 motif-containing sites were only mildly impacted upon AU-15330 treatment in ASCL1-expressing NCI-H69 cells (Figures 2B, S2E, and S2F), suggesting that chromatin accessibility of ASCL1-targeting regions is largely independent of the mSWI/SNF complex. Concurrent with the loss of chromatin accessibility, chromatin immunoprecipitation followed by sequencing (ChIP-seq) showed diminished chromatin binding of POU2F3 and its coactivators (POU2AF2 and POU2AF3) at the AU-15330-mediated loss sites, as examined by tagging endogenous or exogenous POU2F3 and its coactivators in SCLC-P cell lines (Figures 2C and S2G–S2M). Notably, loss of chromatin accesibility and occupancy of POU2F3 and POU2AF2 were detected at 4 h AU-15330 treatment, prior to changes observed in their protein levels (Figures 1E and S1C); this suggests that SMARCA2/4 degradation directly affects physical access of POU2F3 and its coactivators to DNA.

Given the pronounced impact on POU motif-containing sites upon mSWI/SNF complex inactivation, we hypothesized an association between the mSWI/SNF complex and the POU2F3 complex in SCLC-P cells. To explore this, we conducted fast protein liquid chromatography (FPLC) experiments to size fractionate nuclear lysates from two SCLC-P cell lines. We observed several mSWI/SNF complex components (SMARCD1, ARID1A, and SS18), POU2F3, and POU2AF2 co-expressed in the large nuclear fractions (Figure S3A), suggesting a potential coexistence of the POU2F3 complex and the mSWI/SNF complex within a large nuclear protein complex. Further, rapid immunoprecipitation mass spectrometry of endogenous proteins (RIME) analysis of POU2F3 and its coactivators’ interactome revealed multiple key mSWI/SNF components coimmunoprecipitated with POU2F3 and its coactivators (Figures 2D and S3B–S3E, Table S1), affirming the physical association between the POU2F3 complex and the mSWI/SNF complex in SCLC-P cells. Real-time quantitative reverse transcription PCR (RT-qPCR) and global transcriptomic profiling via RNA sequencing (RNA-seq) showcased significant downregulation of *POU2F3*, *POU2AF2/3*, and their downstream targets (e.g., *PTGS1*) in multiple SCLC-P cell lines (Figures 2E and 2F). The gene set enrichment analysis (GSEA) of global AU-15330-mediated transcriptomic alterations reflected a high concordance between mSWI/SNF inactivating gene signatures and transcriptional signatures associated with genetic knockout of *POU2F3* and its co-activators (Figures 2G and S3F).^15^ Additionally, we observed a consistent reduction in ATAC-seq and ChIP-seq signals at several well-established POU2F3 target genes (Figures 2H and S2M). Collectively, our multi-omics analysis suggests that the POU2F3 complex necessitates the mSWI/SNF complex to modulate chromatin accessibility at its DNA binding regions, thereby transactivating the POU2F3 downstream signaling pathway in SCLC-P cells.

### Selective inhibition of SCLC-P xenograft tumor growth by AU-24118

To enhance the translational relevance of our findings, we developed an orally bioavailable SMARCA2/4 PROTAC degrader, named AU-24118, which exhibits enhanced pharmacokinetic (PK) properties compared to AU-15330.^29^ AU-24118 effectively degraded SMARCA2, SMARCA4, and PBRM1, and displayed a preferential growth inhibitory effect for SCLC-P cell lines compared to SCLC-A, SCLC-N, and SCLC-Y cell lines (Figures S3G and S3H). These findings were similar to those shown in Figure S1D for AU-15330, with both SMARCA2/4 degraders inhibiting growth of SCLC-P cells at IC_50_ values in the low nanomolar range.

**Figure 3.**
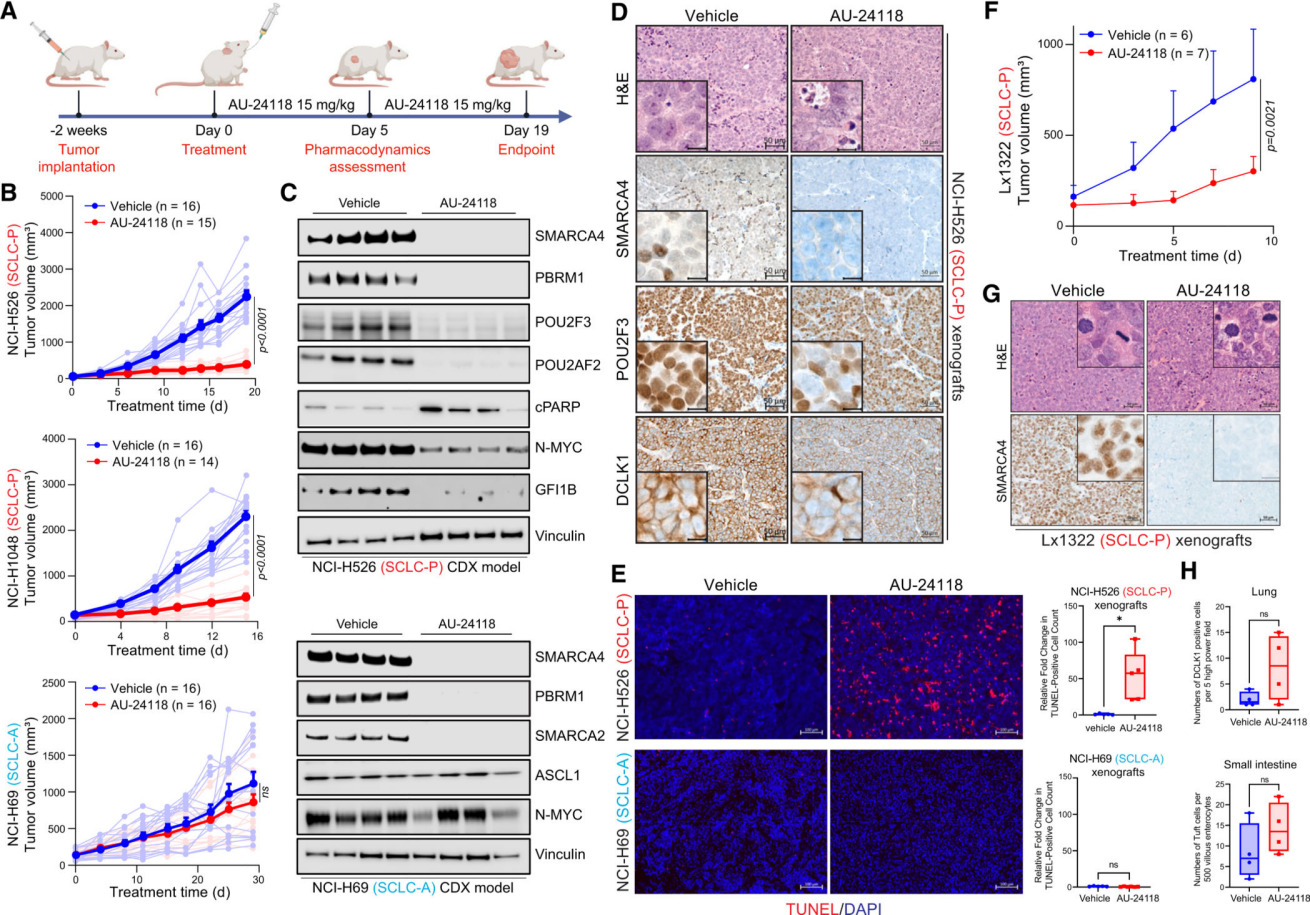
Selective inhibition of SCLC-P xenograft tumor models employing an orally bioavailable mSWI/SNF ATPase degrader (A) Overview of the AU-24118 efficacy study conducted using SCLC xenograft models. (B) Analysis of tumor volume in indicated SCLC xenograft models upon treatment with AU-24118, measured bi-weekly using calipers. Statistical analysis was performed using a two-way ANOVA. Data are presented as mean ± SEM. (C) Immunoblots illustrating levels of the indicated proteins in SCLC-P and SCLC-A xenografts after 5 days of AU-24118 administration. Vinculin is utilized as the loading control across immunoblots. CDX, cell line-derived xenograft. (D) Representative H&E staining with corresponding IHC analyses for SMARCA4, POU2F3, and DCLK1 after 5 days of treatment with AU-24118 in NCI-H526 xenografts (scale, 50 mm). The inset scale, 20 μm. (E) (left) Representative DAPI and TUNEL staining from xenografts from indicated cell lines after 5 days of AU-24118 treatment (scale, 100 μm). (right) Quantitative evaluation of TUNEL staining of respective SCLC xenografts for 5 days. t tests were used to calculate the significance. *p* value < 0.05 in the top panel. The whiskers extend from the minimum to the maximum values, indicating the full range of the data. The middle line represents the median of the data. The box spans from the first quartile (Q1, 25th percentile) to the third quartile (Q3, 75th percentile), representing the interquartile range (IQR). (F) Analysis of tumor volume in Lx1322 patient-derived xenograft (PDX) model upon treatment with AU-24118, measured bi-weekly using calipers. Statistical analysis was performed using a two-way ANOVA. Data are presented as mean ± SEM. (G) Representative H&E staining with corresponding IHC analyses for SMARCA4 after 5 days of treatment with AU-24118 in Lx1322 PDX (scale, 50 mm). The inset scale, 20 μm. (H) DCLK1 cell positivity in lung and small intestine for endpoint evaluation. AU-24118 (15 mg/kg) dosed. Ns, not significant (t tests). The whiskers extend from the minimum to the maximum values, indicating the full range of the data. The middle line represents the median of the data. The box spans from the first quartile (Q1, 25th percentile) to the third quartile (Q3, 75th percentile), representing the interquartile range (IQR). See also Figures S3 and S4.

To define the anti-tumor efficacy of AU-24118 in SCLC, the drug was administered orally at 15 mg/kg, three times weekly, to immunodeficient mice bearing subcutaneous SCLC tumors representing the SCLC-P (NCI-H526 and NCI-H1048) and SCLC-A (NCI-H69) molecular subtypes (Figure 3A). Notably, significant reductions in SCLC-P tumor volumes (Figure 3B) and tumor weights (Figure S3I) were observed post-oral administration of AU-24118. Conversely, AU-24118 treatment did not significantly alter tumor growth of NCI-H69 SCLC-A xenografts (Figures 3B and S3I), thereby confirming the selective anti-tumor efficacy of mSWI/SNF ATPase degraders in SCLC-P preclinical models. Aligning with our observations *in vitro*, SCLC-P tumors treated with AU-24118 exhibited significant degradation of its direct targets (SMARCA2/4 and PBRM1), which ensued in downregulation of POU2F3, POU2F3 coactivators, and downstream target GFI1B (Figure 3C). Additionally, levels of cleaved PARP were increased in SCLC-P tumors treated with AU-24118, while N-MYC levels decreased (Figure 3C). Histopathological assessments performed on AU-24118-treated SCLC-P tumors showed increased apoptotic bodies and intra-tumoral nuclear and necrotic debris in contrast to highly cellular and monotonous appearing, high-grade vehicle-treated tumor samples (Figures 3D and S3J). Fluorometric terminal deoxynucleotidyl transferase (TUNEL) assay analysis confirmed a significant increase in TUNEL-positive cells in SCLC-P but not SCLC-A tumors (Figure 3E). Immunohistochemistry (IHC) further confirmed a dramatic loss of SMARCA4 and POU2F3 protein expression in the AU-24118-treated SCLC-P tumors, as well as decreased DCLK1 expression—a tuft cell marker (Figures 3D and S3J). Despite no changes in tumor growth in the SCLC-A xenografts, immunoblotting and IHC analysis of tumors confirmed on-target drug activity of AU-24118 as indicated by efficient loss of SMARCA4, SMARCA2, and PBRM1 (Figures 3C and S3K).

To further assess the clinical relevance of our findings, we investigated the potential of combining SMARCA2/4 PROTAC degrader treatment with chemotherapy (cisplatin and etoposide), the standard of care for SCLC patients.^30,31^ *In vitro* synergy was assessed between chemotherapy (cisplatin or etoposide) and AU-24118 in multiple SCLC-P cell lines, but results showed no significant synergy with either AU-15330 and cisplatin or AU-15330 and etoposide (Figures S4A–S4C). Potential *in vivo* synergy was next assessed in two SCLC-P xenograft models, evaluating whether AU-24118 could enhance the anti-tumor effects of combined cisplatin and etoposide treatment (Figure S4D). Notably, both *in vivo* studies indicated 10–20% loss in mice body weights in the AU-24118 and chemotherapy combination treated group (Figure S4E), but not with the AU-24118 single agent group, suggesting caution in concurrent administration of both AU-24118 and chemotherapy. Tumor volumes were not significantly different between the AU-24118 and AU-24118 plus chemotherapy treatment groups in the NCI-H1048 xenografts (Figure S4D). Due to the pronounced decrease in mouse body weights with the AU-24118 and chemotherapy combination treatment in the NCI-H526 xenograft study, chemotherapy treatment was stopped at day 8 (Figure S4E). When tumor volumes were followed over time in the NCI-H526 model, addition of cisplatin and etoposide to AU-24118 treatment demonstrated enhanced inhibitory effects compared to AU-24118 or chemotherapy alone, even though chemotherapy was stopped at day 8 (Figure S4D).

Given the potent anti-tumor effects of single agent AU-24118 in cell line-derived xenograft (CDX) models, patient-derived samples were next assessed. Target protein degradation was observed in both patient-derived xenograft (PDX)-derived organoids tested, Lx1322 (SCLC-P) and Lx761C (SCLC-A) (Figure S4F), with improved growth inhibitory effects in Lx1322 compared to Lx761C (Figure S4G). SCLC-P PDX Lx1322 was then used to evaluate the anti-tumor efficacy of AU-24118 *in vivo*. Similar to the findings in the SCLC-P CDX models, AU-24118 significantly inhibited tumor growth in the Lx1322 PDX without any changes in body weight (Figures 3F, 3G, S4H, and S4I).

Lastly, a comprehensive and detailed histopathological assessment showed no remarkable changes or toxic effect with AU-24118 compared to vehicle-treated animals in lung, liver, spleen, kidney, and small intestine tissues with no discernible changes in body weights (Figures S4J and S4K). As POU2F3 and its cofactors are key regulators for the normal tuft cells, IHC for DCLK1 in small intestine and lung tissues of AU-24118 and vehicle-treated mice revealed no statistically significant changes in DCLK1 levels (Figures 3H, S4L, and S4M). Collectively, these results position AU-24118 as an orally bioavailable mSWI/SNF ATPase degrader with potent anti-tumor efficacy and no signs of toxicity as a single agent in preclinical models of the SCLC-P molecular subtype.

### POU2AF1-dependent B cell malignancies exhibit vulnerability to SMARCA2/4 PROTAC degraders

The B cell-specific POU2AF1 coactivator is a paralog of POU2AF2 and POU2AF3 that all share a conserved peptide that binds to the POU2F family of TFs, orchestrating B cell development, maturation, and germinal center formation.^13,32–34^ In diffuse large B cell lymphoma (DLBCL) cells, the *POU2AF1* locus is the most BRD4-overloaded super-enhancer, highlighting its significance in DLBCL growth and other B cell malignancies.^35^ Given the observed functional and physical associations between POU2AF2/3 and the mSWI/SNF complex, we speculated a similar dependency may exist with the POU2AF1 coactivator and the mSWI/SNF complex in B cell malignancies.

**Figure 4.**
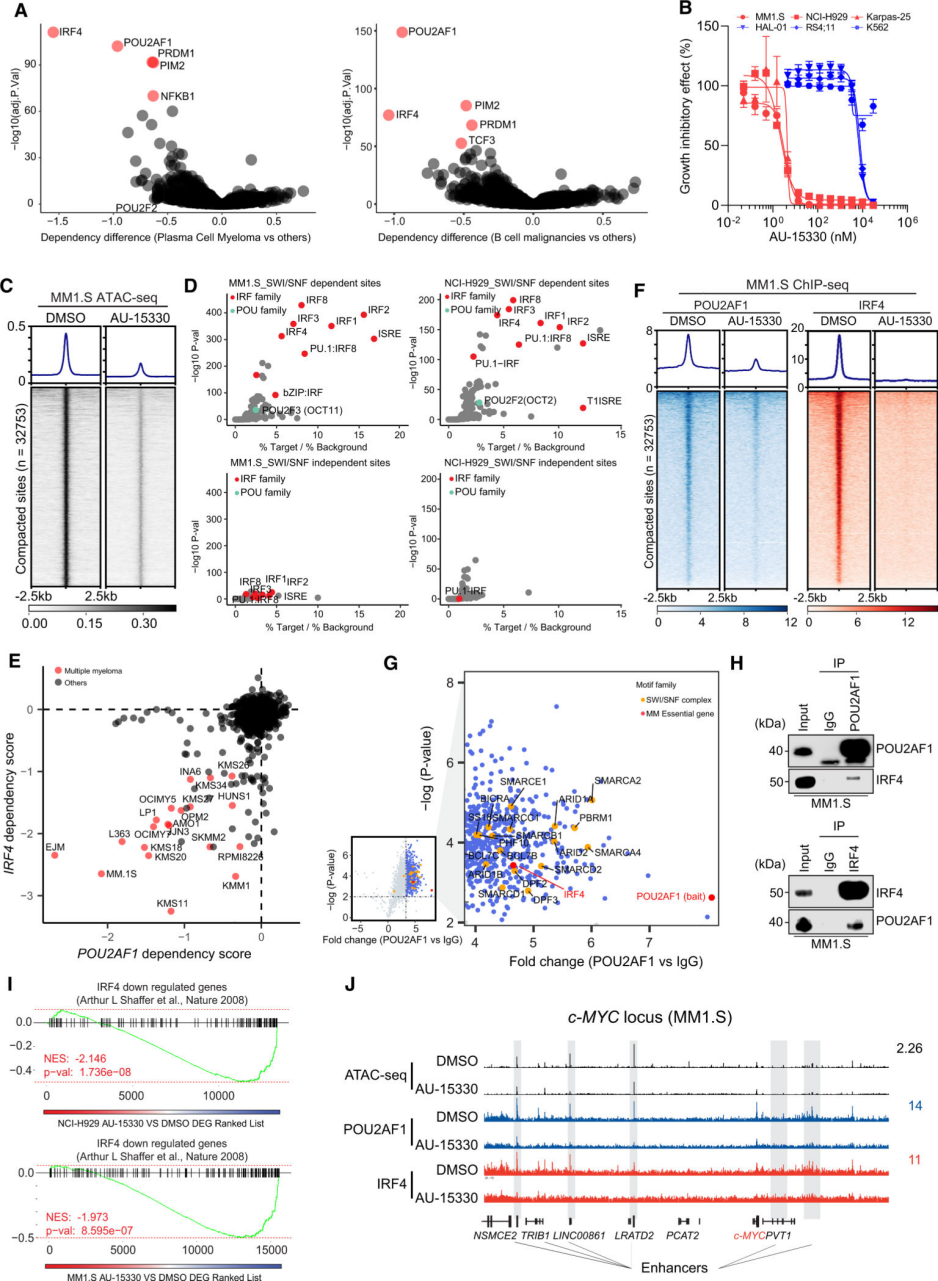
POU2AF1-driven multiple myeloma is dependent on the mSWI/SNF complex (A) Scatterplot depicting gene dependency difference of all plasma cell myeloma versus other cancer types (left) and all B cell malignancies versus other cancer types (right) based on DepMap. The red circles indicate the top 5 essential genes among others. (B) Representative hematological cancer cell lines showing dose-response curves of AU-15330 at varying concentrations for five days. Sensitive cell lines are in red while relatively resistant cell lines are in blue. Data are presented as mean ± SD (*n* = 6). (C) ATAC-seq read-density heatmaps from MM1.S cells treated with DMSO or 1 μM AU-15330 for 4 h (*n* = 2 biological replicates). (D) Analysis of fold change and significance level for HOMER motifs that are enriched within sites dependent and independent of the mSWI/SNF complex after 4 h AU-15330 treatment in MM1.S cells (left panels) and NCI-H929 cells (right panels). (E) Scatterplot showing the dependency scores for IRF4/POU2AF1 in diffuse large B cell lymphoma (blue), multiple myeloma (red), and other cancer types based on DepMap dataset. (F) ChIP-seq read-density heat maps for POU2AF1 and IRF4 at the AU-15330-loss genomic sites in MM1.S cells after treatment with DMSO or AU-15330 (1 μM) for 6 h. (G) Volcano plot detailing proteins that interact with POU2AF1, as identified by POU2AF1 RIME analysis in MM1.S cells. mSWI/SNF components highlighted in orange (*n* = 3 biological replicates). (H) Co-immunoprecipitation (IP) of POU2AF1 or IRF4 in MM1.S cells followed by immunoblot for POU2AF1 and IRF4. This experiment was repeated independently twice. (I) GSEA plots illustrating genes regulated by IRF4. The plots use a gene signature ranked by fold change from AU-15330 treated NCI-H929 (top) and MM1.S (bottom) cells. (J) Combined ATAC-seq and ChIP-seq tracks for *c-MYC* locus in MM1.S cells with and without AU-15330 treatment. See also Figure S5.

Using data from the DepMap project,^36,37^ we confirmed that POU2AF1 is selectively indispensable for the growth of DLBCL and multiple myeloma (MM) cells but not essential in other cancer types (Figure 4A). Given the selective dependency of SCLC-P cells on the mSWI/SNF complex, we investigated whether POU2AF1-dependent B cell malignancies also exhibited sensitivity to SMARCA2/4 degraders. Initially, three MM cell lines tested showed enhanced sensitivity to growth inhibition by AU-15330 compared to three cell lines from other hematological malignancies (Figure 4B). These MM cell lines exhibited rapid loss of targeted proteins (SMARCA4, PBRM1), as well as POU2AF1 and c-MYC at extended time points (Figure S5A). Across an expanded panel of MM and DLBCL cell lines, a subset displayed heightened sensitivity to AU-15330, with IC_50_ values below 200 nM (Figures S5B and S5C), indicating an enhanced dependency on the mSWI/SNF complex.

Experiments were undertaken to define the mechanism of action of mSWI/SNF ATPase degraders in sensitive MM cell lines. Chromatin accessibility changes in two AU-15330 sensitive MM cell lines (MM1.S and NCI-H929) were assessed through ATAC-seq. As observed in SCLC-P cells, AU-15330 decreased genome-wide chromatin accessibility in both tested MM cell lines (Figures 4C, S5D, and S5E). Notably, *de novo* motif analysis of AU-15330-loss sites revealed that, unlike SCLC-P cells, interferon regulatory factor (IRF) motif-containing sites, rather than POU motif-containing sites, were most enriched within AU-15330-loss sites (Figures 4D, S5F, and S5G). Given IRF4’s central role in MM tumorigenesis^38^ and the absence of POU motifs in the MM ATAC-seq data, we postulated that POU2AF1 might act as a transcriptional coactivator of IRF4 by forming a master regulator complex, similar to the relationship between POU2AF2/3 and POU2F3 in SCLC-P cells. Analysis of the DepMap data indicated a significant positive correlation between the essentiality scores of IRF4 and POU2AF1 in MM cells, whereas sole knockout of POU2F1 and POU2F2 were less essential (Figures 4E and S5H). Subsequent ChIP-seq analysis revealed a concordant loss of both POU2AF1 and IRF4 binding within sites affected by AU-15330 (Figures 4F and S5I). Strikingly, *de novo* motif analysis revealed significant enrichment of IRF motifs within POU2AF1 binding sites, suggesting potential formation of a complex containing these regulators at certain genomic loci (Figure S5J). Moreover, RIME experiments confirmed an association between POU2AF1, IRF4, and components of the mSWI/SNF complex in MM1.S cells (Figure 4G). Additionally, reciprocal co-immunoprecipitation experiments validated the IRF4 and POU2AF1 interaction (Figures 4H and S5K). Global transcriptomic profiling via RNA-seq showcased significant downregulation of IRF4 downstream targets^38^ in two MM cell lines treated with AU-15330 (Figures 4I, 4J, and S5L). These data together identify POU2AF1 and IRF signaling as essential regulators of MM cells that are sensitive to inhibition with mSWI/SNF ATPase degraders.

### SMARCA2/4 PROTAC degraders slow tumor growth and increase survival in MM preclinical models

**Figure 5.**
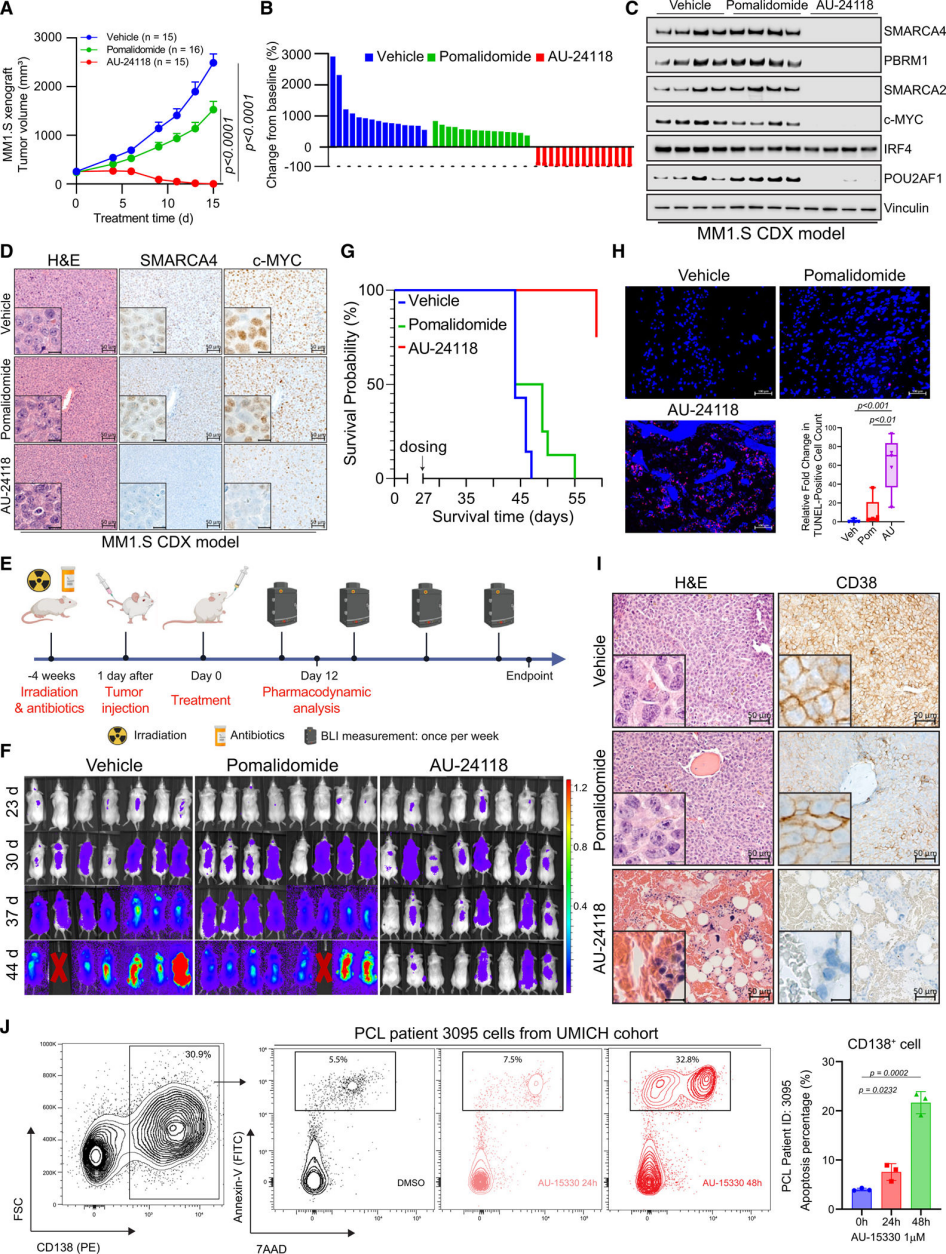
Potent tumor inhibition is induced by mSWI/SNF ATPase degraders in various preclinical multiple myeloma models (A) Analysis of tumor volumes in the MM1.S xenograft model upon treatment with AU-24118 and pomalidomide, measured bi-weekly using calipers. Statistical analysis was performed using a two-way ANOVA. Data are presented as mean ± SEM. (B) Waterfall plot depicting change in tumor volume at the study endpoint for MM1.S-derived xenograft models. (C) Immunoblot illustrating levels of the indicated proteins in MM1.S xenografts after AU-24118 treatment for 5 days. Vinculin is utilized as the loading control. (D) Representative H&E staining with corresponding IHC analyses for SMARCA4 and c-MYC after 5 days of the indicated treatment in MM1.S xenografts (scale, 50 mm). The inset scale, 20 μm. (E) Overview of the MM1.S multiple myeloma disseminated xenograft model efficacy study. (F) Bioluminescent images of MM1.S disseminated xenograft model after different treatments. Mice were monitored once per week. The signal intensity of bioluminescence represented the tumor burden (x10^8^ photons/sec/cm^2^/steradian). Pomalidomide (10 mg/kg) and AU-24118 (15 mg/kg) dosed. (G) Kaplan-Meier survival curve of MM1.S disseminated xenograft model after pomalidomide (10 mg/kg) and AU-24118 (15 mg/kg) treatment. (H) Representative DAPI and TUNEL staining from the MM1.S disseminated xenograft model and quantitative evaluation from TUNEL staining for pomalidomide (10 mg/kg) and AU-24118 (15 mg/kg) treatment for 12 days. The whiskers extend from the minimum to the maximum values, indicating the full range of the data. The middle line represents the median of the data. The box spans from the first quartile (Q1, 25th percentile) to the third quartile (Q3, 75th percentile), representing the interquartile range (IQR). (I) Representative H&E and CD38 IHC staining of spinal vertebral marrow after *in vivo* administration of pomalidomide (10 mg/kg) and AU-24118 (15 mg/kg) for 12 days. (J) Quantification of flow cytometry measuring apoptosis signal in DMSO, 24 h or 48 h with 1 μM AU-15330 in CD138 positive cells (top) or CD138 negative cells (bottom) in fresh plasma cell leukemia (PCL) patient cells. The same patient (3095) bulk cell population data was used in Figure S7A. t tests were used to calculate the significance. Data are presented as mean ± SD (*n* = 3). See also Figures S6 and S7.

To evaluate the therapeutic potential of targeting the mSWI/SNF ATPases in MM, we evaluated the anti-tumor efficacy of the orally bioavailable degrader, AU-24118, across diverse MM preclinical models. Initially, immunodeficient mice bearing MM subcutaneous tumors (MM1.S, NCI-H929, and Karpas-25) were treated with either vehicle, pomalidomide (10 mg/kg, p.o., five times weekly), carfilzomib (5 mg/kg, i.v., bi-weekly), or AU-24118 (15 mg/kg, p.o., three times weekly) (Figure S6A). In all three models, AU-24118 significantly decreased tumor volumes and weights compared to pomalidomide or carfilzomib, without notable alterations in body weights (Figures 5A, 5B, and S6B–S6F). Notably, tumor regression was observed in all animals treated with AU-24118 in the MM1.S xenograft study (Figures 5A and 5B). Western blot analysis confirmed targeted protein degradation (SMARCA2, SMARCA4, and PBRM1) and downregulation of c-MYC and POU2AF1 in MM1.S tumors treated with AU-24118 (Figure 5C). Histopathological evaluation further supported the efficacy of AU-24118 treatment, with marked loss of SMARCA4 and downregulation of c-MYC (Figure 5D). A disseminated orthotopic xenograft model of MM was next used to more physiologically recapitulate the disease state in patients. Luciferase and green fluorescent protein (GFP) dual-expressing MM1.S cells were injected into mice via the tail vein four weeks after irradiation (Figures 5E and S6G). Vehicle, pomalidomide, or AU-24118 were then orally administered. The luciferase signal showed a substantial reduction over time and at endpoint, indicative of diminished tumor proliferation (Figures 5F, S6H, and S6I). A notable extension in the overall survival of mice treated with AU-24118 was observed (Figure 5G), and TUNEL staining was significantly increased following AU-24118 treatment (Figure 5H). IHC confirmed loss of SMARCA4 and c-MYC exclusively in AU-24118-treated tumors in the MM1.S disseminated model (Figure S6J).

Histopathological evaluation of orthotopic xenografts to assess the efficacy of the mSWI/SNF ATPase degrader was undertaken (Figure 5I). Pathological assessment revealed that in comparison to the vehicle where sheets of plasma cells were noted, there was an absence of any perceptible plasma cells in the AU-24118-treated group. Additionally, in AU-24118-treated tumors, we identified remnant hematopoietic cells intermixed (not seen in vehicle tumor tissues) with a fair number of red blood cell (RBC)-filled sinusoidal areas. The presence of areas filled with RBCs in the sinusoids in the marrow tissue, which appear to be areas of drug-mediated tumor regression, along with the presence of hematopoietic cells, provides additional direct (*in situ*) biological evidence of the efficacy of our degrader (Figure 5I). This was in turn validated molecularly with CD38 IHC, where, in comparison to diffuse strong membranous positivity of CD38 in all marrow cells of the vehicle tumor tissue, there was near total absence of CD38 in any remnant cells in the AU-24118-treated orthotopic xenografts. This points toward a significant and complete abatement of tumor cells upon AU-24118 treatment. Additionally, a standard of care therapeutic (pomalidomide) showed some depletion of plasma cells but not a degree of depletion as seen in the AU-24118-treated group at both morphological and molecular levels (Figure 5I).

The anti-cancer efficacy of SMARCA2/4 degraders was next evaluated with *ex vivo* patient-derived cells from cases of plasma cell leukemia (PCL), an aggressive form of MM, and chronic myelogenous leukemia (CML). Flow cytometry analysis demonstrated selective induction of apoptosis in plasma cells following AU-15330 treatment, while BCL-ABL fusion-driven CML cells remained unaffected (Figures 5J and S7A–S7C). Morphological evaluation via Diff-Quik staining and molecular confirmation through immunocytochemistry (ICC) demonstrated loss of SMARCA4 protein in AU-15330 treated plasma cells (Figures S7D and S7E). AU-15330 exhibited potent growth inhibitory effects in cells derived from PCL compared to cells derived from CML (Figure S7F). Immunoblotting analysis confirmed that AU-15330 induced effective target protein degradation (SMARCA4 and SMARCA2) and downregulation of c-MYC, POU2AF1, and IRF4 and induction of cleaved PARP in PCL cells (Figure S7G). Consequently, leveraging patient-derived cells, our study underscores the potential translational impact of targeting the mSWI/SNF complex with PROTAC degraders, particularly in POU2AF1/IRF4-dependent MM.

## DISCUSSION

Transcription factors are frequently dysregulated in the pathogenesis of human cancer, representing a major class of cancer cell dependencies. Targeting these factors can significantly impact the treatment of specific malignancies, as exemplified by the clinical success of agents targeting the androgen receptor (AR) in prostate cancer and estrogen receptor (ER) in breast cancer.^39^ Conventional small-molecule drugs exert their effects by binding to defined pockets on target protein surfaces, such as the ligand binding domains of AR and ER. However, many TFs lack structurally ordered ligand binding pockets, presenting significant challenges in therapeutically targeting their actions. As an alternative strategy, targeting of TF coregulators has emerged as a promising approach to block their functions in cancer.^40^ We previously found that inhibiting the mSWI/SNF chromatin remodeling complex disrupts oncogenic signaling of key TFs (AR, FOXA1, ERG, and MYC) in castration-resistant prostate cancer (CRPC).^23^ Here, we identify the mSWI/SNF complex as a therapeutic vulnerability in other TF-driven malignancies, namely POU2F3-driven SCLC and POU2AF1-dependent B cell malignancies. Importantly, we show that an orally bioavailable mSWI/SNF ATPase degrader, AU-24118, has anti-tumor activity in multiple preclinical models of both SCLC-P and MM with no signs of toxicity.

Our study reveals a significant reliance of SCLC-P cells, distinct from other molecular subtypes, on the mSWI/SNF complex, highlighting its pivotal role in regulating POU2F3 signaling. The unique dependency of SCLC-P cells on the mSWI/SNF complex is attributed to the physical interaction between the POU2F3-POU2AF2/3 complex and the mSWI/SNF complex. The findings also suggest further investigation into the mechanisms governing ASCL1’s transcriptional activity in SCLC-A cells as ASCL1 may rely on alternative mechanisms to modulate chromatin accessibility in SCLC-A cells. In addition to SMARCA2 and SMARCA4, our research identified several sgRNAs targeting other mSWI/SNF components which were significantly enriched in SCLC-P cells, including BRD9. This aligns with findings from a genome-scale positive selection screen that underscored BRD9 as an essential regulator of POU2F3.^41^ The mSWI/SNF complex critically relies on its ATPase subunits, SMARCA2/4, for chromatin remodeling functions; thus, their degradation could impede the functions of all mSWI/SNF complex variants, such as canonical BAF (cBAF), polybromo-associated BAF (pBAF), and non-canonical BAF (ncBAF) complexes. Targeting BRD9, a key component of the ncBAF complex, may provide a selective therapeutic strategy for a subset of SCLC-P cells, potentially broadening the therapeutic window owing to their retention of canonical mSWI/SNF complex function. Furthermore, we explored the combination of SMARCA2/4 degraders with chemotherapy, the standard of care treatment for SCLC patients. Although no significant synergy was observed *in vitro*, we noted significant enhancement of anti-tumor efficacy in the chemotherapy naive NCI-H526 CDX model. However, concurrent treatment with chemotherapy and AU-24118 requires caution due to observed animal weight loss. Notably, AU-24118 monotherapy demonstrated significant efficacy in an SCLC-P PDX model derived from a patient who had relapsed on chemotherapy, highlighting its promising therapeutic potential in treatment regimens for SCLC that is refractory to chemotherapy. Lastly, SCLC shares transcriptional drivers with neuroendocrine prostate cancer (NEPC),^42^ and the mSWI/SNF complex has been suggested to be involved in NEPC.^43^ Recently, multiple single-cell analyses have identified a subpopulation of NEPC cells with high expression of POU2F3 and its downstream target ASCL2 in both prostate cancer patients and genetically engineered mouse models (GEMMs).^44–47^ As androgen deprivation therapy (ADT) continues to be a standard treatment for prostate cancer, the emergence of NEPC post-ADT underscores the need to explore mSWI/SNF targeting therapies in POU2F3-expressing NEPC.

We also demonstrate that mSWI/SNF ATPase degraders possess potent therapeutic activity against subsets of MM and DLBCL cells reliant on POU2AF1. Typically, POU2AF1 functions as coactivator of the POU2 family of transcription factors, pivotal in orchestrating B cell development and the tumorigenesis of B cell malignancies. Our multi-omics analysis has uncovered a previously unidentified role for POU2AF1 as a coactivator for IRF4, in addition to its known interactions with POU2F1 (OCT-1) and POU2F2 (OCT-2). POU2AF1 enhances IRF4’s regulatory functions, forming a complex analogous to the POU2AF2/3 and POU2F3 interaction in SCLC-P cells. Previous studies have also shown that POU2AF1’s chromatin binding significantly overlaps with other transcription factors, including c-MYC and IRF4, underscoring its critical role in transcriptional regulation in MM cells.^37^ Building on this, our findings reveal that mSWI/SNF ATPase degrader treatment markedly diminishes chromatin accessibility at IRF4 binding regions in MM cells, evicting both IRF4 and POU2AF1 from DNA, thereby impeding IRF4-mediated oncogenic transcriptional activity. These results are consistent with the observed robust anti-tumor effects of SMARCA2/4 degraders in various MM preclinical models. Additionally, we observed that SMARCA2/4 degraders effectively inhibit the growth of a subset of DLBCL cells, which may be attributed to POU2AF1’s dependence on the mSWI/SNF complex. A similar phenotype has been reported in ARID1A-mutant lymphoma cells,^48^ suggesting further investigation will be needed to clarify the mechanism of action of SWI/SNF-targeting therapeutics in DLBCL. Considering IRF4’s critical role in B cell malignancies and the absence of FDA-approved therapies that directly target IRF4, our study provides significant insight, offering an alternative therapeutic approach by targeting the mSWI/SNF complex and impeding the function of the POU2AF1 coactivator.

The embryonic lethality observed upon genetic knockout of the ATPase subunit of the mSWI/SNF complex necessitates a thorough examination of the toxicity profile associated with ATPase subunit degradation *in vivo*.^49,50^ Our *in vivo* assessments with the orally bioavailable SMARCA2/4 PROTAC degrader, AU-24118, demonstrated a favorable tolerability profile alongside significant anti-tumor efficacy in multiple SCLC-P and MM preclinical models. Moreover, in the *in vivo* models of SCLC-P, AU-24118 treatment did not affect tuft cells in normal tissues. Effective regenerative processes were also observed in disseminated orthotopic xenograft models of MM, addressing concerns regarding potential adverse effects on normal cellular processes. Similar observations were made by Papillon et al., where hematopoietic stem cells (HSC) isolated from BRM014 (SMARCA2/4 inhibitor)^51^-treated mice retained their functionality, suggesting transient loss of mSWI/SNF function does not permanently suppress HSC function.^25^ Recent studies delineating the role of the mSWI/SNF complex in memory T cell fate suggest that modulating mSWI/SNF activity early in T cell differentiation can enhance cancer immunotherapy outcomes,^52,53^ thereby warranting future studies to evaluate the anti-tumor efficacy and safety of mSWI/SNF-targeting strategies in syngeneic tumor models in immunocompetent mice.

Collectively, this study identifies the mSWI/SNF chromatin remodeling complex as a vulnerability in POU2F3-dependent SCLC and POU2AF1-dependent MM. Combined with our previous findings in CRPC,^23^ these findings position mSWI/SNF ATPase degraders as potential candidates for further optimization and clinical testing across various cancer types, reinforcing the value of TF co-regulator targeting strategies in oncology.

## STAR★METHODS

### RESOURCE AVAILABILITY

#### Lead contact

Further information and requests for resources should be directed to and will be fullfilled by the lead contact, Arul M. Chinnaiyan (arul@med.umich.edu).

#### Materials availability

All materials used in this paper are available from the lead contact upon request.

#### Data and code availability

ATAC-seq, ChIP-seq, and RNA-seq data have been deposited at the Gene Expression Omnibus (GEO), and the accession number is listed in the key resources table. This paper does not report original code. Any additional information required to reanalyze the data reported in this work is available from the lead contact upon request.

### EXPERIMENTAL MODEL AND STUDY PARTICIPANT DETAILS

#### Cell lines

All cell lines were originally obtained from ATCC, DSMZ, ECACC, or internal stock. All cell lines were genotyped to confirm their identity at the University of Michigan Sequencing Core and tested biweekly for mycoplasma contamination. NCI-H526, NCI-H1048, NCI-H211, COR-L311, MM1.S, and Karpas-25 were grown in Gibco RPMI-1640 + 10% fetal bovine serum (FBS) (Thermo Fisher Scientific). NCI-H929 was grown in Gibco RPMI-1640 + 10% FBS + 0.05 mM 2-mercaptoethanol. All cell lines were cultured at 37°C in incubators with 5% CO_2_ atmosphere.

#### POU2F3/AF2/AF3-dTAG-HA system expressing SCLC cells

For HA-dTAG-POU2F3 or POU2AF2/3-dTAG-HA system, the FKBP23F36V-2xHA was PCR amplified from the pCRIS-PITCHv2-Puro-dTAG vector (Addgene: 91793)^56^ and introduced into sgRNA-resistant POU2F3_LentiV_neo or the POU2AF2/3_LentiV_neo vector for functional validation with competition-based cell proliferation assay.^55^ NCI-H1048/NCI-H526 that stably expressed Cas9 were infected either with HA_dTAG_POU2F3_LentiV_neo or POU2AF2/3_dTAG_HA_LentiV_neo or empty_vector_lentiV_neo construct followed by neomycin selection to establish stable cell lines. The cells were then lentivirally delivered with indicated sgRNAs co-expressed with a GFP reporter. The percentage of GFP^+^ cells correspond to the sgRNA representation within the population. GFP measurements in human cell lines were taken on day 4 post-infection and every four days with Guava Easycyte HT instrument (Millipore). The fold change in GFP+ population (normalized to day 4) was used for analysis. HA_dTAG_POU2F3 or POU2AF2/3_dTAG_HA, which is resistant to its own sgRNA, were cloned into the LRGB2.1T vector (Addgene: 108098) that either contains sgRNA against endogenous POU2F3 or POU2AF2/3 into NCIH211/NCIH526/NCIH1048 that stably express Cas9.^55^

#### Human tumor xenograft models

Six-week-old CB17 severe combined immunodeficiency (SCID) mice were procured from the University of Michigan breeding colony. The gender of the mice used in each experiment was matched to the gender of the patient from which the cell line orginated. All mice were randomly assigned to vehicle and experimental groups. Subcutaneous tumors were established at both sides of the dorsal flank of mice before starting treatment. Tumors were measured at least biweekly using digital calipers following the formula (π/6) (L × W^2^), where L is length and W is width of the tumor. The disseminated model was measured by signal intensity of luminescence by PerkinElmer’s IVIS Spectrum from the University of Michigan Imaging Core. At the end of the studies, mice were killed and tumors extracted and weighed. The University of Michigan Institutional Animal Care and Use Committee (IACUC) and the Memorial Sloan Kettering Cancer Center (MSKCC) Animal Care and Use Committee approved all *in vivo* studies. For the NCI-H526, NCI-H1048, and NCI-H69 models, 5 × 10^6^ tumor cells were injected subcutaneously into the dorsal flank on both sides of the mice in a serum-free medium with 50% Matrigel (BD Biosciences). Once tumors reached a palpable stage (~100 mm^3^), mice were randomized and then treated with either 15 mg kg^−1^ AU-24118 or vehicle by oral gavage 3 days per week for 3 – 4 weeks, and with or without 1 mg kg^−1^ cisplatin 1 day per week and 1 mg kg^−1^ etoposide 3 days per week by intraperitoneal injection. For the NCI-H929 and MM1.S models, 5 × 10^6^ cells were injected subcutaneously into the dorsal flank on both sides of the mice in a serum-free medium with 50% Matrigel (BD Biosciences). Once tumors reached a palpable stage (~100 mm^3^), mice were randomized and then treated with the following as indicated in the figures: 15 mg kg^−1^ AU-24118 by oral gavage 3 days per week, 10 mg kg^−1^ pomalidomide by oral gavage 5 days per week, 5 mg kg^−1^ carfilzomib by intravenous administration for two consecutive days and 5 days rest, or vehicle for 3–4 weeks. For the Karpas-25 tumor model, 3 × 10^6^ cells were injected subcutaneously into the dorsal flank on both sides of the mice in a serum-free medium with 50% Matrigel (BD Biosciences). Once tumors reached a palpable stage (~100 mm^3^), mice were randomized and then treated with either 15 mg kg^−1^ AU-24118 by oral gavage 3 days per week, 5 mg kg^−1^ carfilzomib by intravenous injection for two consecutive days injection and 5 days rest, or vehicle for 3–4 weeks. For the MM1.S disseminated model, 1 × 10^7^ GFP/luc MM1.S cells were injected intravenously from the tail vein of the mice in a PBS medium after 24 hours 250 cGy r-irradiation using the Small Animal Radiation Research Platform (SARRP). The mice were then treated with 1 mg/ml neomycin water bottle for 3 weeks in case of infection due to irradiation. Once the signal of luminescence reached a measurable stage (~1 × 10^6^), mice were randomized and then treated with either 15 mg kg^−1^ AU-24118 by oral gavage 3 days per week, pomalidomide by oral gavage 5 days per week, or vehicle until the mice reached the endpoint based on protocol. For the Lx1322 patient-derived model, 2 × 10^6^ tumor cells after dissociation were injected subcutaneously into the dorsal flank on both sides of the NOD.Cg-Prkdc<scid>Il2rg<tm1Wjl>/SzJ (Stock #: 005557) (6–8 weeks old) mice obtained from Jackson Laboratory in a serum-free medium with 50% Matrigel (BD Biosciences). Once tumors reached a palpable stage (~100 mm^3^), mice were randomized and then treated with either 15 mg kg^−1^ AU-24118 or vehicle by oral gavage 3 days per week for 2 weeks. Following the IACUC guidelines, in all treatment arms, the maximal tumor size did not exceed the 2.0 cm limit in any dimension, and animals with xenografts reaching that size were duly euthanized.

#### SCLC PDX model

Patient samples for the generation of PDX models and subsequent analyses were collected with written informed consent from patients under protocols approved by the MSKCC Institutional Review Board/Privacy Board. The details about Lx1322 and Lx761C were described previously.^72,73^

#### GFP/Luc MM1.S cell line

MM1.S cells were transduced with GFP luciferase lentivirus (purchased from the Vector Core of University of Michigan) through spinfection (45 minutes at 600g). Two days after viral transduction, the GFP-positive cells were sorted with a cell sorter (SONY SH800S).

#### Patient information and ethics

Patient samples were obtained from plasma cell leukemia (PCL) and chronic myelogenous leukemia (CML) patients after informed consent approved by the University of Michigan Institutional Review Board, Ann Arbor, MI, USA. Patient information is described in Table S2.

### METHOD DETAILS

#### CRISPR screening library generation

The paralog co-targeting CRISPR library was generated to use SpCas9, a system recently published.^74^ Oligonucleotide pools, targeting 4,341 single genes and 4,387 paralogs using 137,950 double guide RNAs, were synthesized (Twist Bioscience) and cloned into LRG3.0,^59^ a lentiviral vector with human U6 and bovine U6 promoters expressing the two sgRNAs in inverse orientation. Cas9 stable cell lines were transduced with Cas9 vector (Addgene: 108100).^55^ Cell lines were transduced with the paralog co-targeting CRISPR library virus to achieve a representation of 1,000 cells per sgRNA at a low multiplicity of infection (around 0.3). SCLC cell lines were transduced while spun for 45 min at 600g. On day 6 after transduction, cells were selected using blasticidin, split, and replated to maintain representation. An initial sample was taken using the remainder. Once 10 cell doublings were reached, cells were pelleted by centrifugation and frozen, or genomic DNA was extracted directly.

#### Genomic DNA extraction

Cells were resuspended in resuspension buffer (10 mM Tris-HCl pH=8.0, 150 mM NaCl, 10 mM EDTA) with the addition of proteinase K (0.02 mg/mL) and SDS (final concentration 0.1%). Lysate was incubated at 56°C for 48h. Genomic DNA was extracted using two rounds of TRIS-saturated phenol (Thermo Fisher Scientific) extraction.

#### dgRNA PCR for illumina sequencing

For PCR from genomic DNA, 1 μg of genomic DNA was used for each reaction. In round 1, PCR with 11 cycles was used. DNA was purified using a gel extraction kit (QIAGEN) according to the manufacturer’s instructions. Product DNA was barcoded by amplification in a second round PCR using stacked P5/P7 primers. PCR products were again purified and sequenced on NextSeq with the paired-end 75 base pair (bp) reads protocol (Illumina). Reads were counted by mapping the pairs of 19–20 nt sgRNAs to the reference sgRNA list containing combinations present in the library. 16 pseudo counts were added prior to downstream analysis. The resulting matrix of read counts was used to calculate log2 fold changes.

#### Cell viability assay

Cells were plated onto 96-well plates in their respective culture medium and incubated at 37°C in an atmosphere of 5% CO_2_. After overnight incubation, a serial dilution of compounds was prepared and added to the plate. The cells were further incubated for 5 days, and the CellTiter-Glo assay (Promega) was then performed according to the manufacturer’s instruction to determine cell proliferation. The luminescence signal from each well was acquired using the Infinite M1000 Pro plate reader (Tecan), and the data were analyzed using GraphPad Prism software (GraphPad Software).

#### Western blot

Western blot was performed as previously described.^23^ In brief, cell lysates were prepared in RIPA buffer (Thermo Fisher Scientific) supplemented with protease inhibitor cocktail tablets (Sigma-Aldrich). Total protein concentration was measured by Pierce BCA Protein Assay Kit (Thermo Fisher Scientific), and an equal amount of protein was loaded in NuPAGE 3 to 8% Tris-Acetate Protein Gel (Thermo Fisher Scientific) or NuPAGE 4 to 12% Bis-Tris Protein Gel (Thermo Fisher Scientific) and blotted with primary antibodies. Following incubation with HRP-conjugated secondary antibodies, membranes were imaged on an Odyssey CLx Imager (LiCOR Biosciences). Antibody details are described in the key resources table and Table S3.

#### RNA isolation and quantitative real-time PCR

Total RNA was isolated from cells using the Direct-zol kit (Zymo), and cDNA was synthesized using Maxima First Strand cDNA Synthesis Kit for PCR with reverse transcription (RT–PCR) (Thermo Fisher Scientific). Quantitative real-time PCR (qPCR) was performed in triplicate using standard SYBR green reagents and protocols on a QuantStudio 7 Real-Time PCR system (Applied Biosystems). The target mRNA expression was quantified using the ΔΔCt method and normalized to *ACTB* expression. Primer sequences are listed in the key resources table.

#### ATAC-seq

ATAC-seq was performed as previously described.^75^ In brief, cells treated with AU-15330 were washed in cold PBS and resuspended in RSB buffer with NP-40, Tween-20, protease inhibitor and digitonin cytoplasmic lysis buffer (CER-I from the NE-PER kit, Thermo Fisher Scientific). This single-cell suspension was incubated on ice for 5 min. The lysing process was terminated by the addition of double volume RSB buffer with Tween-20. The lysate was centrifuged at 1,300g for 5 min at 4°C. Nuclei were resuspended in 50 μl of 13 TD buffer, then incubated with 0.5–3.5 μl Tn5 enzyme for 30 min at 37°C (Illumina Tagment DNA Enzyme and Buffer Kit; cat. no. 20034198). Samples were immediately purified by Qiagen minElute column and PCR-amplified with the NEB Next High-Fidelity 2X PCR Master Mix (cat. no. M0541L) following the original protocol. qPCR was used to determine the optimal PCR cycles to prevent over-amplification. The amplified library was further purified by Qiagen minElute column and SPRI beads (Beckman Coulter, cat. no. A63881). ATAC-seq libraries were sequenced on the Illumina HiSeq 2500 or NovaSeq.

#### RNA-seq

RNA-seq libraries were prepared using 800 ng of total RNA. PolyA+ RNA isolation, cDNA synthesis, end-repair, A-base addition, and ligation of the Illumina indexed adapters were performed according to the TruSeq RNA protocol (Illumina). Libraries were size selected for 350–500 bp cDNA fragments by using AMPure beads- (65/20 ratio) and using 2x KAPA Hifi HotStart mix and NEB dual indexes for PCR-amplification. Library quality was measured on an Agilent 2100 Bioanalyzer for product size and concentration. Paired-end libraries were sequenced with the Illumina HiSeq 2500 or NovaSeq, (2 × 150 nucleotide read length) with sequence coverage to 15–20M paired reads.

#### ChIP–seq

Chromatin immunoprecipitation (ChIP) experiments were carried out using the ideal ChIP-seq kit for TFs (Diagenode) as per the manufacturer’s protocol. Chromatin from 2 × 10^6^ cells was used for each ChIP reaction with 4 μg of the target protein antibody. In brief, cells were trypsinized and washed twice with 13 PBS, followed by cross-linking for 10 min in 1% formaldehyde solution. Crosslinking was terminated by the addition of 1/10 volume 1.25 M glycine for 5 min at room temperature followed by cell lysis and sonication (Bioruptor, Diagenode), resulting in an average chromatin fragment size of 200 bp. Fragmented chromatin was then used for immunoprecipitation using various antibodies, with overnight incubation at 4°C. ChIP DNA was de-crosslinked and purified using the standard protocol. Purified DNA was then prepared for sequencing as per the manufacturer’s instructions (Illumina). 1–20 ng ChIP DNA samples were end repaired and A-tailed, then ligated with NEB adapters, following by 2xKAPA HiFi HotStart mix and NEB dual indexes PCR to enrich fragments between 200–500 bp. Libraries were quantified and quality checked using the Bioanalyzer 2100 (Agilent) and sequenced on the Illumina HiSeq 2500 or NovaSeq Sequencer (125-nucleotide read length).

#### FPLC

NCI-H526/COR-L311 nuclear extracts were obtained using NE-PER nuclear extraction kit (Thermo Fisher Scientific) and dialyzed against FPLC buffer (20 mM Tris-HCl, 0.2 mM EDTA, 5 mM MgCl2, 0.1 M KCl, 10% (v/v) glycerol, 0.5 mM DTT, 1 mM benzamidine, 0.2 mM PMSF, pH7.9). 5 mg of nuclear protein was concentrated in 500 μl using a Microcon centrifugal filter (Millipore) and then applied to a Superose 6 size exclusion column (10/300 GL GE Healthcare) pre-calibrated using the Gel Filtration HMW Calibration Kit (GE Healthcare). 500 ml elute was collected for each fraction at a flow rate of 0.5ml/min, and eluted fractions were subjected to SDS-PAGE and western blotting.

#### RIME

RIME experiments were carried out as previously described.^76^ In brief, 40 × 10^6^ cells were used for each RIME reaction with 20 μg of the target protein antibody. Cells were harvested followed by cross-linking for 8 min in 1% formaldehyde solution. Crosslinking was terminated by adding glycine to a final concentration of 0.1 M for 5 min at room temperature. Cells were washed with 1x PBS and pelleted by centrifugation at 2000g for 3 min at 4°C for 4 times total. Cell pellets were added to the nuclear extraction buffer LB1, LB2, and LB3 separately. Lysates were sonicated (Bioruptor, Diagenode) to result in an average chromatin fragment size of 200–600 bp. Fragmented nuclear lysates were then used for immunoprecipitation using various antibodies, with overnight incubation at 4°C. All antibodies were preincubated with beads for 1 hour at room temperature. Total protein per replicate was labeled with TMT isobaric Label Reagent (Thermo Fisher Scientific) according to the manufacturer’s protocol and subjected to liquid chromatography—mass spectrometry (LC—MS)/MS analysis.

#### Co-immunoprecipitation

Immunoprecipitations were conducted in HEK293FT and MM1.S cells. HEK293FT cells were transiently transfected with POU2AF1-HA and IRF4-Flag with Lipofectamine 3000 (Thermo Fisher; L300001) based on the manufacturer’s instructions. POU2AF1-HA and IRF4-Flag constructs were directly ordered from Vector Builder and verified with Sanger sequencing by Eurofin Genomics (Louisville, Kentucky). Cell lysates were prepared in Pierce IP lysis buffer (Thermo Fisher Scientific) supplemented with protease inhibitor cocktail tablets (Sigma-Aldrich). The cell lysates were sonicated and centrifuged 10 mins with maximum speed. The supernatant was pre-cleared by Dynabeads Protein G (Thermo Fisher; 10004D) for 2 hours at 4°C. 1% input sample was removed. Lysates were incubated with HA-tag, Flag-tag, IRF4, or POU2AF1 antibody overnight at 4°C. The next day, Dynabeads Protein G were added and incubated for 2 hours at 4°C. Next, beads were washed 4 times with IP lysis buffer, and proteins were eluted. Western blot immunoblotting was then performed as described above.

#### Drugs formula for *in vivo* studies

AU-24118 was added in PEG200 and then sonicated and vortexed until completely dissolved. Five volumes of 10% D-a-Tocopherol polyethylene glycol 1000 succinate was next added, and the solution was vortexed until homogeneous. Four volumes of 1% Tween 80 was then added, and the solution was vortexed until homogeneous. AU-24118 was freshly prepared right before administration to mice. Pomalidomide was dissolved in DMSO and then added in 30% PEG400 + 2% Tween-80 + 68% ddH_2_O. AU-24118 and pomalidomide were delivered to mice by oral gavage. Carfilzomib was diluted in sterile water based on the company’s instructions (Kyprolis). Cisplatin was diluted in 0.9% sodium chloride. Etoposide was dissolved in DMSO and then added in 40% PEG300 + 5% Tween 80 + 45% 0.9% sodium chloride.

#### Histopathological analysis for drug toxicity

For the present study, organs (liver, spleen, kidney, small intestine, and lung) were harvested and fixed in 10% neutral buffered formalin followed by embedding in paraffin to make tissue blocks. These blocks were sectioned at 4 μm and stained with Harris haematoxylin and alcoholic eosin-Y stain (both reagents from Leica Surgipath), and staining was performed on a Leica autostainer-XL (automatic) platform. The stained sections were evaluated by two different pathologists using a brightfield microscope in a blinded fashion between the control and treatment groups for general tissue morphology and coherence of architecture. A detailed comprehensive analysis of the changes noted at the cellular and subcellular level were performed as described below for each specific tissue. Evaluation of liver: Liver tissue sections were evaluated for normal architecture, and regional analysis for all three zones was performed for inflammation, necrosis, and fibrosis. Evaluation of spleen: Splenic tissue sections were evaluated for the organization of hematogenous red and lymphoid white pulp regions including necrosis and fibrotic changes, if any. Evaluation of kidney: Kidney tissue sections were examined for changes noted, if any, in all four renal functional components, namely glomeruli, interstitium, tubules, and vessels. Evaluation of small intestine: Small intestine tissue sections were examined for mucosal changes such as villous blunting, villous: crypt ratio, and evaluated for inflammatory changes including intraepithelial lymphocytes, extent (mucosal, submucosal, serosal), and type of inflammatory infiltrate including tissue modulatory effect. Evaluation of lung: Lung tissue sections were thoroughly examined to identify the presence of regenerative/degenerative atypia in the alveolar and bronchiolar epithelium, hyperplasia of type II pneumocytes, and interstitial pneumonia. The presence of extensive alveolar damage, organized pneumonia (also known as bronchiolitis obliterans organizing pneumonia or BOOP), and alveolar hemorrhage and histology suggesting usual interstitial pneumonitis (UIP) was also investigated. A mild and within normal range proliferation of type II pneumocytes (devoid of other associated inflammatory and other associative findings) was considered within unremarkable histology.

#### Immunohistochemistry and immunocytochemistry

Immunohistochemistry (IHC) was performed on 4-micron formalin-fixed, paraffin-embedded (FFPE) tissue sections using POU2F3, BRG1 (a surrogate marker for SMARCA4), CD38, and DCLK1. IHC was carried out on the Ventana ULTRA automated slide staining system using the Omni View Universal DAB detection kit. The antibody and critical reagent details are provided in the key resources table and Table S3. Either the presence or absence of BRG1 and POU2F3 nuclear staining and DCLK1 and CD38 cytoplasmic/membranous staining were recorded by the study pathologists. To provide a semi-quantitative score per biomarker, a product score was rendered wherever needed. The IHC product score calculated out of 300 was derived by multiplying the percentage of positive tumor cells (PP) for each staining intensity (I) and adding the values in each tumor using the formula ‘‘IHC Score = (PP * 0 + PP * 1 + PP * 2 + PP * 3)’’ as previously described.^77^

Immunocytochemistry (ICC) was performed on cytospin smears fixed with cold acetone (−20°C) on the Ventana ULTRA automated slide staining system using the reagents described above. During the process, the antigen retrieval step was omitted and primary antibody incubation was done under an exteded period at 37°C followed by the ULTRAView detection system.

#### TUNEL assay

Apoptosis was examined using Terminal dUTP Nick End Labeling (TUNEL) performed with an *In Situ* Cell Death Detection Kit (TMR Red #12156792910; Roche Applied Science) following the manufacturer’s instructions. Briefly, fixed sections were permeabilized with Triton X-100, followed by a PBS wash. The labeling reaction was performed at 37°C for 60 min by addition of a reaction buffer containing enzymes. Images were acquired on a Zeiss Axiolmager M1 microscope.

#### Flow cytometry

Mononuclear cells of plasma cell leukemia (PCL) and chronic myelogenous leukemia (CML) patients’ samples were separated from whole blood by Ficoll density-gradient centrifugation and cryopreserved. Before analysis, all samples were thawed and seeded in RPMI-1640 medium and treated in six well plates as indicated. Cells were washed and resuspended in MACS buffer (PBS containing 2% FBS and 2 mM EDTA). CD138 (Miltenyi Biotec; 130–118-840) was stained for the PCL samples following the manufacturer’s protocol. Cells were washed in binding buffer and stained for Annexin-V (BD; 556570) and 7AAD (Thermo Fisher; 00–6993-50) separately. Finally, cells were subjected to flow cytometry assessment (SONY SH800S).

#### Cytospin

Cells were resuspended in PBS containing 0.1% BSA and then centrifuged at 800 rpm for 3 minutes. Slides were air dried or fixed with acetone overnight for further staining.

### QUANTIFICATION AND STATISTICAL ANALYSIS

#### Paralog gene identification and functional domain mapping

Paralog pairs within the human genome were identified using BlastP. Matches of isoforms originating from the same gene were removed. Each individual gene’s top paralog identified (E-value < 0.01) that shared the same functional domain of interest was included in the Paralog library. In addition, each paralog pair was included for genes with multiple high-scoring paralogs (E-value < 10–100). Functional domains were mapped using reverse spi blast (rps-Blast) and the conserved domain database (CDD).^78^

#### Selection of sgRNAs and controls

Domain annotation and sgRNA cutting codon were compared, and sgRNAs cutting in functional domain regions were included in the sgRNA selection pool. sgRNAs with off-targets in paralog genes were removed from the selection pool. sgRNAs were chosen based on their off-target score (calculated based on the number of off-target locations in the human genome and number of miss-matches). For each gene, 3–4 selective domain-focused sgRNA were chosen. In cases in which selective domain-focused targeting sgRNA were not available, sgRNAs targeting the upstream coding region of the gene were selected. For each given paralog pair (A-B), 3–4 sgRNA for paralog A were combined with 3–4 sgRNAs for paralog B, resulting in 9–16 combinations. To evaluate single-gene knockout effects of each gene, each of the paralog’s sgRNA was also combined with each one targeting- and one non-targeting-negative control. A set of known essential genes as positive controls (dgRNA n=28) and a set of non-targeting (dgRNA n=100) as well as non-coding region targeting negative controls (dgRNA n=54) were generated. To construct cell line-specific negative controls (non-synergistic pairs), we selected genes that were not expressed in a cell line according to the RNA sequencing (RNA-seq) data (log2(TPM + 1) < 0.1) from the CCLE.

#### Calculation of paralog CRISPR screening Log_2_ fold changes and synergy scores

Synergy scores were calculated using the GEMINI R package^79^ (Table S1). Briefly, GEMINI calculates the log-fold changes (LFCs) of the sgRNA pair abundance between the initial- and the 10-doubling time endpoint. GEMINI has been used to compute the synergy score by comparing the LFC of each gene pair to the most lethal individual gene of the pair. GEMINI uses non-synergistic pairs to calculate the FDR and p-value in each cell line, as described previously.^79^ Beta scores for single and double knockouts were calculated using MAGeCK^26,79^ and compared between 3 SCLC-A and 3 SCLC-P cell lines. Gene-level beta scores for synergistic double gene knockouts (synergy score > 1) (n=968) and single knockouts were plotted.

#### Genomic alterations in SWI/SNF genes

Somatic mutation data for small cell lung cancer (SCLC) were obtained from a prior study.^54^ Patients were classified into four groups—ASCL1, POU2F3, NEUROD1, and YAP1—based on RNA expression levels. The genomic alterations in SWI/SNF genes were visualized using ComplexHeatmap (version 2.10.0).^66^

#### ATAC-seq analysis

Fastq files were trimmed using Trimmomatic (version 0.39) and then uniquely aligned to the GRCh38/hg38 human genome assembly using bwa mem (version 0.7.17-r1198-dirty) and converted to binary files using SAMtools (version 1.9).^67–69^ Reads mapped to mitochondrial or duplicated reads were removed by SAMtools and PICARD MarkDuplicates (version 2.26.0–1-gbaf4d27-SNAPSHOT), respectively. Filtered alignment files from replicates were merged for downstream analysis. MACS2 (2.1.1.20160309) was used to call ATAC-seq peaks.^59^ UCSC’s tool wigtoBigwig was used for conversion to bigwig formats.^60^ All *de novo* and known motif enrichment analyses were performed using the HOMER (version v4.11.1) suite of algorithms.^61^ *De novo* motif discovery and enrichment analysis of known motifs were performed with findMotifsGenome.pl (–size given). Using the R package ChIPpeakAnno (version 3.0.0), comparisons between samples determined the sites present in DMSO but lost upon AU15330 treatment.^71^ These reduced accessibility sites were then plotted as read density heatmaps using deepTools.^66^

#### RNA-seq analysis

Libraries passing quality control were trimmed of sequencing adapters and aligned to the human reference genome, GRCh38. Samples were demultiplexed into paired-end reads using Illumina’s bcl2fastq conversion software v2.20. The reference genome was indexed using bwa (version 0.7.17-r1198-dirty), and reads were pseudoaligned onto the GRCh38/hg38 human reference genome using Kallisto’s quant command.^63,65^ EdgeR (version 3.39.6) was used to compute differential gene expression using raw read-counts as input.^69^ Limma-Voom (limma_3.53.10) was then used to perform differential expression analysis.^68^ Heatmaps were generated using the ComplexHeatmap package in R. These gene signatures were used to perform a fast pre-ranked GSEA using fgsea bioconductor package in R (version fgsea_1.24.0).^78^ We used the function fgsea to estimate the net enrichment score and p-value of each pathway, and the plotEnrichment function was used to plot enrichment for the pathways of interest.

#### ChIP-seq analysis

Paired-end, 125 bp reads were trimmed and aligned to the human reference genome (GRC h38/hg38) with the Burrows-Wheeler Aligner (BWA; version 0.7.17-r1198-dirty) The SAM file obtained after alignment was converted into BAM format using SAMTools (version 1.9).^69^ Picard MarkDuplicates command and samtools were used to filter aligned output. MACS2 (version 2.1.1.20160309) callpeak was used for performing peak calling with the following option: ‘macs2 callpeak–call-summits–verbose 3 -g hs -f BAM -n OUT–qvalue 0.05.^70^ Blacklisted regions of the genome were removed using bedtools. UCSC’s tool wigtoBigwig was used for conversion to bigwig formats. ChIP peak profile plots and read-density heatmaps were generated using deepTools, and cistrome overlap analyses were carried out using the ChIPpeakAnno (version 3.0.0) or ChIPseeker (version 1.29.1) packages in R (version 3.6.0).^73,74,79^

#### IHC scoring for normal organs

To rule out modulatory effects on the molecular levels as predicted by unremarkable morphology on histopathological assessment of the normal organs, a specialized histology score was devised to fit the individual organ systems. For the intestine, the number of DCLK1-positive cells/ 500 intestinal enterocytes (predominantly villi of small intestine) were counted; for lung parenchyma, the number of DCLK1-positive cells/5 high power fields were counted.

## Supplementary Material

1

2

Supplemental information can be found online at https://doi.org/10.1016/j.ccell.2024.06.006.

## Figures and Tables

**Table:** KEY RESOURCES TABLE

REAGENT or RESOURCE	SOURCE	IDENTIFIER

**Antibodies**		

SMARCA2/BRM	Bethyl Laboratories	Cat#A301–016A; RRID: AB_2193933
SMARCA4/BRG1	Cell Signaling Technology	Cat# 52251S; RRID: AB_2799410
PBRM1	Bethyl Laboratories	Cat# A301–591A; RRID: AB_1078808
Vinculin	Cell Signaling Technology	Cat# 18799S; RRID: AB_2714181
c-Myc	Abcam	Cat# ab32072; RRID: AB_731658
Cleaved PARP (Asp214)	Cell Signaling Technology	Cat# 9541; RRID: AB_331426
POU2F3	Cell Signaling Technology	Cat# 92579
ASCL1	Abcam	Cat# ab74065; RRID: AB_1859937
POU2AF2	Cell Signaling Technology	Cat# 20217s
N-Myc	Santa Cruz	Cat# sc-53993; RRID: AB_831602
GFI1B	Santa Cruz	Cat#sc-28356; RRID: AB_2110132
NEUROD1	Abcam	Cat#ab60704; RRID: AB_943491
HA-tag	Cell Signaling Technology	Cat# 3724s; RRID: AB_1549585
SMARCD1	Santa Cruz	Cat#sc-135843; RRID: AB_2192137
POU2AF1	Cell Signaling Technology	Cat#43079s
IRF4	Cell Signaling Technology	Cat# 4964s; RRID: AB_10698467
ARID1A	Santa Cruz	Cat# sc-373784; RRID: AB_10917727
DCLK1	Abcam	Cat# ab109029; RRID: AB_10864128
BRG1	Abcam	Cat# ab108318; RRID: AB_10889900
POU2F3	Cell Signaling Technology	Cat# mAB#36135/clone E5N2D; RRID: AB_2924784
CD38	Ventana	Cat# 760–4785/clone SP149
POU2AF1	Thermo Fisher Scientific	Cat# PA5–121026; RRID: AB_2914598
CD138	Miltenyi Biotec	Cat# 130–119-840; RRID: AB_2751879

Bacterial and virus strains		

One Shot^™^ Stbl3^™^ Chemically Competent *E. coli*	Invitrogen	Cat#C737303

Biological samples		

Human plasma cell leukemia cells 9527	This paper	Table S2
Human plasma cell leukemia cells 0823	This paper	Table S2
Human plasma cell leukemia cells 3095	This paper	Table S2
Human chronic myelogenous leukemia cells CML-L1	This paper	Table S2
Human chronic myelogenous leukemia cells CML-L3	This paper	Table S2

Chemicals, peptides, and recombinant proteins		

AU-15330	Aurigene	N/A
AU-24118	Aurigene	N/A
Pomalidomide	Selleck Chemicals	Cat#S1567
Carfilzomib	Kyprolis	N/A
Etoposide	Selleck Chemicals	Cat#S1225
Cisplatin	Selleck Chemicals	Cat#S1166
NP-40	Thermo Scientific	Cat#85125
Tween-20	Millipore Sigma	Cat#11332465001
Digitonin	Fisher Scientific	Cat#PRG9441
NEB Next High-Fidelity 2X PCR Master Mix	New England Biolabs	Cat#M0541L
Qiagen minElute column and SPRI beads	Beckman Coulter	Cat#A63881
T4 DNA ligase	New England Biolabs	Cat#M0202L
KAPA RNA Hyper+RiboErase HMR	Roche Diagnostics	Cat#08098140702
NEBNExt Multiplex Oligos for Illumina	New England Biolabs	Cat#E6440L
KAPA Hyper Prep	Kapa Biosystems	Cat#KK8504
FastDigest AscI	Thermo Scientific	Cat#FD1894
FastDigest 119I	Thermo Scientific	Cat#FD0124
Formaldehyde	Sigma Aldrich	Cat#F8775
Dynabeads Protein G	Thermo Scientific	Cat#10004D
Lipofectamine^™^ 3000 Transfection Reagent	Invitrogen	Cat#L3000001
Puromycin	Thermo Scientific	Cat#A1113803
Blasticidin	Thermo Scientific	Cat#A1113903
Fast SYBR^™^ Green Master Mix	Thermo Scientific	Cat#4385612
IP lysis buffer	Thermo Scientific	Cat#87788

Critical commercial assays		

CellTiter-Glo^®^ Luminescent Cell Viability Assay	Promega	Cat#G7572
TMTpro^™^ 16plex Label Reagent Set	Thermo Scientific	Cat#A44521
iDeal ChIP-seq Kit for Transcription Factors	Diagenode	Cat#C01010170
Illumina Tagment DNA Enzyme and Buffer Large Kit	Illumina	Cat#20034198
Direct-zol RNA Purification Kits	Zymo Research	Cat#R2052
Maxima First Strand cDNA Synthesis Kit for RT-qPCR	Thermo Scientific	Cat#K1641
*In Situ* Cell Death Detection kit, TMR red	Sigma Aldrich	Cat#12156792910

Deposited data		

Raw and analyzed data	This paper	GEO: GSE247951
WES and RNA-seq data of SCLC cohort	Liu et al.^54^	GSA database: HRA003419

Experimental models: Cell lines		

NCI-H526	ATCC	RRID: CVCL_1569
NCI-H1048	ATCC	RRID: CVCL_1453
NCI-H211	ATCC	RRID: CVCL_1529
COR-L311	Millipore Sigma	RRID: CVCL_2412
MM1.S	ATCC	RRID: CVCL_8792
NCI-H929	ATCC	RRID: CVCL_1600
Karpas-25	Sigma Aldrich	RRID: CVCL_2540

Experimental models: Organisms/strains		

CB17/*Icr-Prkdcscid*/IcrIcoCrl	Charles River	RRID:IMSR_CRL:236
NOD.Cg-Prkdcscid Il2rg	The Jackson Laboratory	RRID:IMSR_JAX:005557

Oligonucleotides		

POU2F3 Fwd: CCAGTGCCCAAGCATCTACC	Wu et al.^15^	N/A
POU2F3-Rev: GTCGTTGCCATACAGCTTTCC	Wu et al.^15^	N/A
POU2AF2-Fwd: AGACTACAGCAAACGAGTGTATC	Wu et al.^15^	N/A
POU2AF2-Rev: GGAACTGACGCTGCCATTA	Wu et al.^15^	N/A
POU2AF3-Fwd: CTTTAACCAGAGCCTGATCCC	Wu et al.^15^	N/A
POU2AF3-Rev: ACTGTAGTCTAAGGAGCCAGAG	Wu et al.^15^	N/A
PTGS1-Fwd: CGCCAGTGAATCCCTGTTGTT	This paper	N/A
PTGS1-Rev: AAGGTGGCATTGACAAACTCC	This paper	N/A
ACTB_Fwd: AGGATGCAGAAGGAGATCACTG	This paper	N/A
ACTB_Rev: AGTACTTGCGCTCAGGAGGAG	This paper	N/A

Recombinant DNA		

LentiV_Cas9_puro	Tarumoto et al.^55^	Addgene Plasmid #108100
pCRIS-PITChv2-Puro-dTAG	Nabet et al.^56^	Addgene Plasmid #91793
hPGK-POU2AF1-HAx3-Puro	Vectorbuilder	N/A
gblock: IRF4-FLAG	IDT	N/A
LRG3.0	Klingbeil et al.^57^	N/A
LRG2.1T	Tarumoto et al.^55^	Addgene Plasmid #108098
lentiV_P2A_Neo	Tarumoto et al.^55^	Addgene Plasmid #108101

Software and algorithms		

ComplexHeatmap	Gu et al.^58^	https://bioconductor.org/packages/release/bioc/html/ComplexHeatmap.html
MACS2 (2.1.1.20160309)	Zhang et al.^59^	https://pypi.org/project/MACS2/
UCSC wigtoBigwig	Kent et al.^60^	https://www.encodeproject.org/software/wigtobigwig/
HOMER (version v4.11.1)	Heinz et al.^61^	http://homer.ucsd.edu/homer/index.html
Trimmomatic (version 0.39)	Bolger et al.^62^	
bwa (version 0.7.17-r1198-dirty)	Li et al.^63^	
PICARD MarkDuplicates (version 2.26.0–1-gbaf4d27-SNAPSHOT)	N/A	https://broadinstitute.github.io/picard/
SAMtools (version 1.9)	Danecek et al.^64^	https://www.htslib.org/doc/1.9/samtools.html
Kallisto (0.46.1)	Bray et al.^65^	https://pachterlab.github.io/kallisto/manual
Deeptools (3.5.1)	Ramirez et al.^66^	https://deeptools.readthedocs.io/en/develop/
R (version 3.6.0)	N/A	N/A
fgsea (version fgsea_1.24.0)	Korotkevich et al.^67^	https://bioconductor.org/packages/release/bioc/html/fgsea.html
Limma-Voom (limma_3.53.10)	Ritchie et al.^68^	https://bioconductor.org/packages/release/bioc/html/limma.html
EdgeR (version 3.39.6)	Robinson et al.^69^	https://bioconductor.org/packages/release/bioc/html/edgeR.html
ChIPseeker (version 1.29.1)	Yu et al.^70^	https://guangchuangyu.github.io/software/ChIPseeker/
ChIPpeakAnno (version 3.0.0)	Zhu et al.^71^	https://bioconductor.org/packages/release/bioc/html/ChIPpeakAnno.html

## Highlights

- mSWI/SNF complex and its ATPase subunit are epigenetic dependencies in SCLC-P
- POU2F3 complex requires the mSWI/SNF complex to modulate chromatin accessibility
- POU2AF1 and IRF4 function as an epigenetic complex requiring mSWI/SNF in MM
- An oral mSWI/SNF ATPase degrader shows potent anti-tumor efficacy in SCLC-P and MM
