# Supplementary material for: Targeting the mSWI/SNF complex in POU2F-POU2AF transcription factor-driven malignancies
Source: Cancer Cell. Author manuscript; Available in PMC 2025 Jun 9. (PMC12147762; doi:10.1016/j.ccell.2024.06.006)
Supplement: 1 [file NIHMS2073889-supplement-1.pdf]

Cancer Cell, Volume *42*

Supplemental information

Targeting the mSWI/SNF complex in POU2F-POU2AF transcription factor-driven malignancies

Tongchen He (贺彤琛), Lanbo Xiao (肖兰博), Yuanyuan Qiao (乔源远), Olaf Klingbeil, Eleanor Young, Xiaoli S. Wu (吴小丽), Rahul Mannan, Somnath Mahapatra, Esther Redin, Hanbyul Cho, Yi Bao (鲍奕), Malathi Kandarpa, Jean Ching-Yi Tien (田静宜), Xiaoju Wang, Sanjana Eyunni, Yang Zheng (郑洋), NamHoon Kim, Heng Zheng, Siyu Hou (侯思宇), Fengyun Su, Stephanie J. Miner, Rohit Mehra, Xuhong Cao, Chandrasekhar Abbineni, Susanta Samajdar, Murali Ramachandra, Saravana M. Dhanasekaran, Moshe Talpaz, Abhijit Parolia, Charles M. Rudin, Christopher R. Vakoc, and Arul M. Chinnaiyan

Figure S1

A

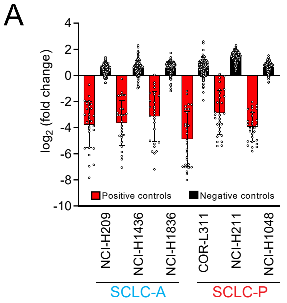

C

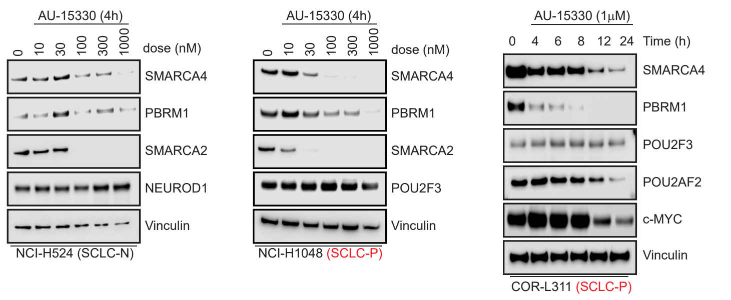

D

SCLC cell line	Subtype	AU-15330 (nM)
NCI-H526	SCLC-P	1.8
COR-L311	SCLC-P	8.86
NCI-H1048	SCLC-P	11.45
NCI-H211	SCLC-P	21.91
NCI-H69	SCLC-A	4406
NCI-H1963	SCLC-A	5275
NCI-H209	SCLC-A	8075
COR-L47	SCLC-A	10278
NCI-H1436	SCLC-A	12795
SW1271	SCLC-Y	25460
NCI-H82	SCLC-N	>30000
NCI-H446	SCLC-N	>30000
NCI-H524	SCLC-N	>30000
NCI-H1836	SCLC-A	>30000
HCC-33	SCLC-N	>30000

B

sgRNA combinations	sgRNA number	SCLC-P COR-L311	SCLC-P NCI-H1048	SCLC-P NCI-H211	SCLC-A NCI-H1436	SCLC-A NCI-H1836	SCLC-A NCI-H209	Δbeta score
sgSMARCA2 (Bromodomain) + sgControl	8	-1.19	0.07	-1.09	-0.03	-0.18	-0.4	-0.54
sgSMARCA4 (Bromodomain) + sgControl	8	-1.83	-0.73	-2.12	-0.2	-0.26	-0.33	-1.29
sgSMARCA2 (Bromodomain) + sgSMARCA4 (Bromodomain)	16	-2.13	-1.00	-2.28	-0.37	-0.7	-0.66	-1.22
sgSMARCA2 (RRM domain) + sgControl	8	-0.39	-0.08	-0.73	0.32	-0.09	-0.05	-0.46
sgSMARCA4 (RRM domain) + sgControl	8	-2.76	-1.39	-2.14	-0.88	-0.66	-0.71	-1.35
sgSMARCA2 (RRM domain) + sgSMARCA4 (RRM domain)	16	-2.14	-1.19	-2.31	-1.05	-0.92	-1.24	-0.81
sgSMARCA2 (Bromodomain) + sgPBRM1 (Bromodomain)	16	-1.62	-0.24	-1.39	-1.11	-0.73	-0.77	-0.21
sgBRD9 (Bromodomain) + sgControl	8	-2.64	-0.49	-1.89	-0.36	-0.51	-1.09	-1.02
sgBRD9 (Bromodomain) + sgBRD7 (Bromodomain)	12	-3.32	-1.15	-2.17	-0.59	-1.03	-1.54	-1.16

E

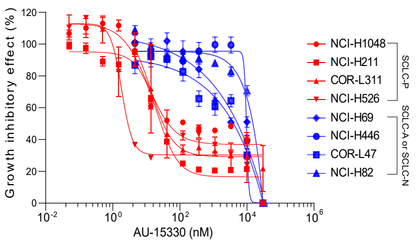

F

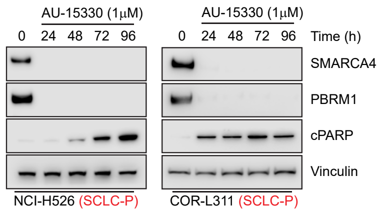

G

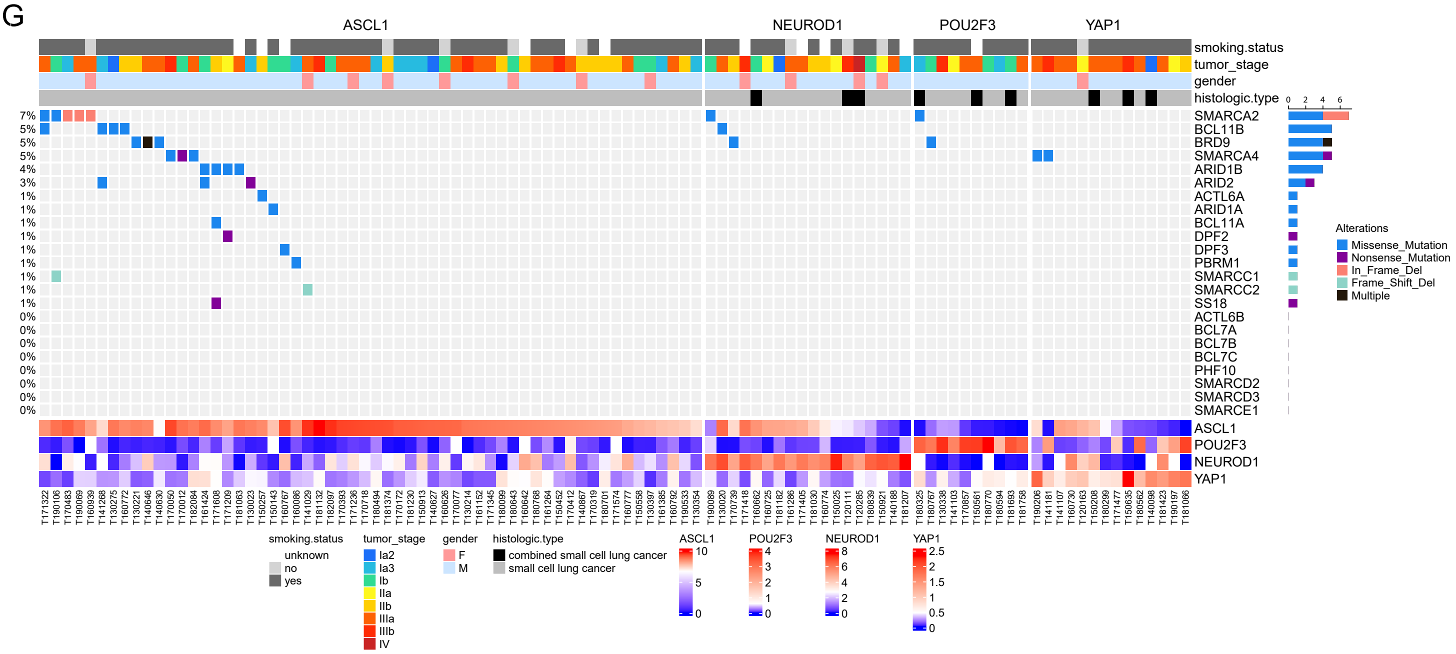

H

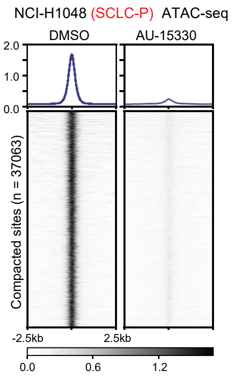

I

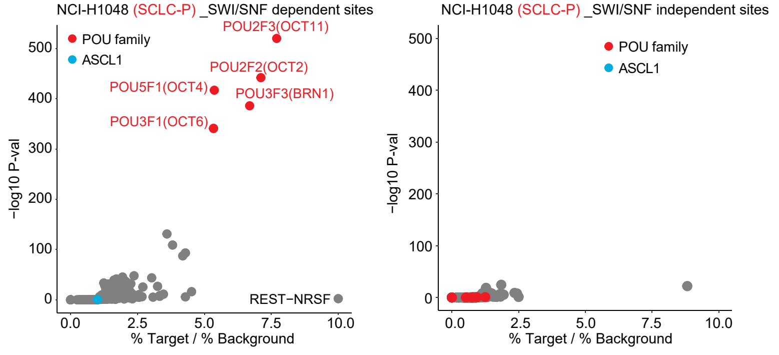

Figure S1. Selective essentiality of the mSWI/SNF complex in the POU2F3 molecular subtype of SCLC and loss of chromatin accessibility with mSWI/SNF ATPase degrader treatment. Related to Figures 1-2.

(A) Fold change of positive and negative controls in CRISPR screen. Data are presented as mean +/- SD.

(B) Beta score for different domains of druggable targeted gene in mSWI/SNF complex in SCLC-P cell lines versus SCLC-A cell lines. RRM, RNA recognition motif.

(C) Immunoblot analysis of indicated proteins in SCLC cells post-treatment with varying time points (right) or concentrations (left two experiments, four hours) of AU-15330. Vinculin serves as the control for protein loading in all immunoblots.

(D) IC_{50} value of five days of AU-15330 treatment for different subtypes of SCLC cell lines.

(E) Representative dose-response curves of SCLC-P and SCLC-A cells treated with AU-15330 at varying concentration for five days. Data are presented as mean +/- SD (n = 6).

(F) Immunoblot analysis verifying apoptosis in SCLC-P cells post-treatment with varying time points of AU-15330. Vinculin serves as the control for protein loading in all immunoblots.

(G) Genomic mutational landscape of the CPTAC SCLC patients (n = 100). Upper, clinical information; middle, heatmap for driver mutations; lower, RNA expression (log2 TPM+1) of *ASCL1*, *POU2F3*, *NEUROD1*, and *YAP1*. (p = 0.0705) Analyzed with Chi-Squared test.

(H) Reduced chromatin accessibility induced by mSWI/SNF ATPase degradation. Visualization of ATAC-seq read-density in NCI-H1048 (SCLC-P) post-treatment for 4 hours with either vehicle or 1 μM AU-15330 (n = 2 biological replicates).

(I) Analysis of fold change and significance level for HOMER motifs that are enriched within sites dependent and independent of the mSWI/SNF complex in NCI-H1048 cells.

Figure S2

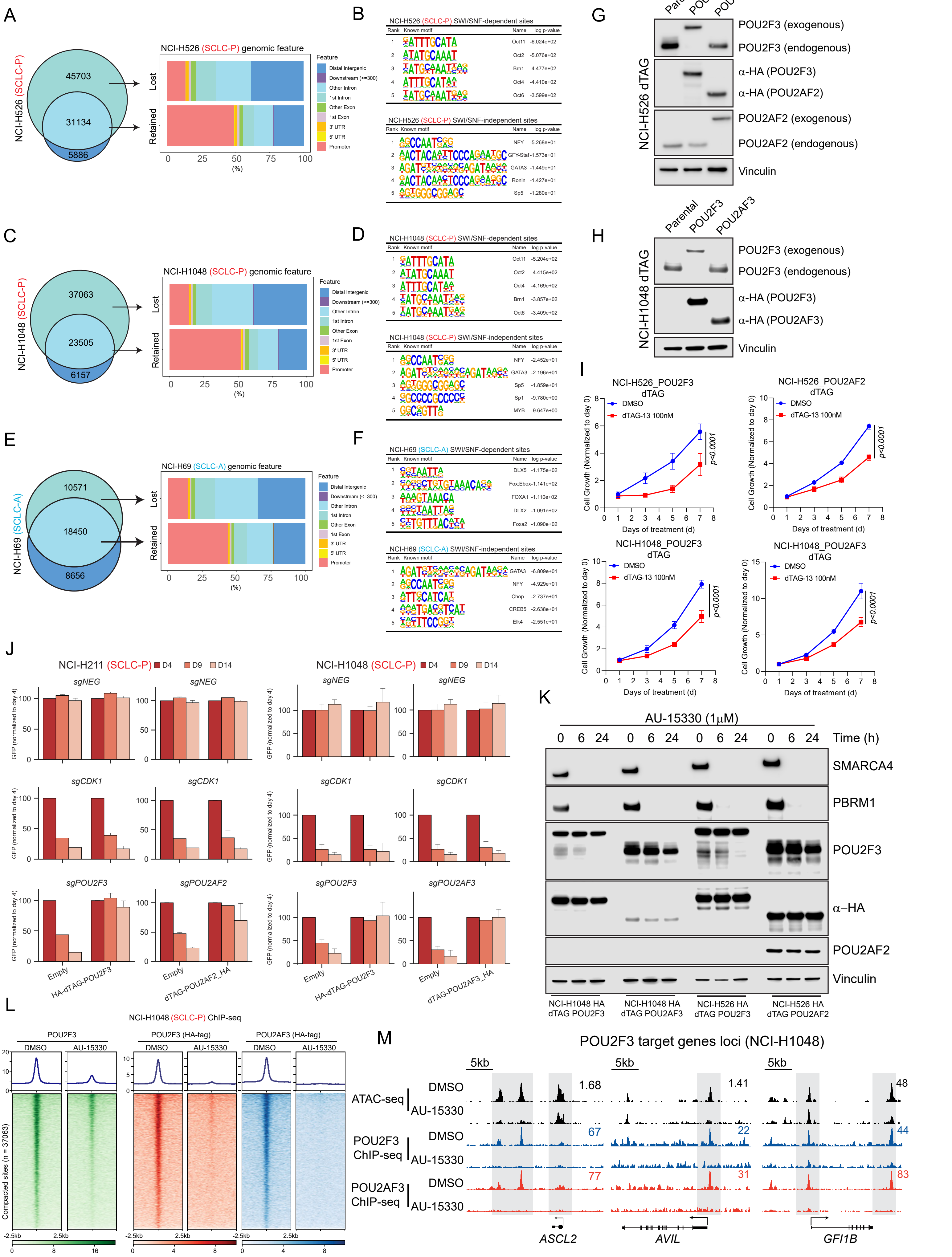

A
NCI-H526 (SCLC-P)
45703
31134
5886
NCI-H526 (SCLC-P) genomic feature
Lost
Retained
Feature
Distal Intergenic
Downstream (<=300)
Other Intron
1st Intron
Other Exon
1st Exon
3' UTR
5' UTR
Promoter
B
NCI-H526 (SCLC-P) SWI/SNF-dependent sites
NCI-H526 (SCLC-P) SWI/SNF-independent sites
C
NCI-H1048 (SCLC-P)
37063
23505
6157
NCI-H1048 (SCLC-P) genomic feature
D
NCI-H1048 (SCLC-P) SWI/SNF-dependent sites
NCI-H1048 (SCLC-P) SWI/SNF-independent sites
E
NCI-H69 (SCLC-A)
10571
18450
8656
NCI-H69 (SCLC-A) genomic feature
F
NCI-H69 (SCLC-A) SWI/SNF-dependent sites
NCI-H69 (SCLC-A) SWI/SNF-independent sites
G
NCI-H526 dTAG
Parental
POU2F3
POU2AF2
POU2F3 (exogenous)
POU2F3 (endogenous)
α-HA (POU2F3)
α-HA (POU2AF2)
POU2AF2 (exogenous)
POU2AF2 (endogenous)
Vinculin
H
NCI-H1048 dTAG
Parental
POU2F3
POU2AF3
POU2F3 (exogenous)
POU2F3 (endogenous)
α-HA (POU2F3)
α-HA (POU2AF3)
Vinculin
I
NCI-H526_POU2F3 dTAG
NCI-H526_POU2AF2 dTAG
NCI-H1048_POU2F3 dTAG
NCI-H1048_POU2AF3 dTAG
DMSO
dTAG-13 100nM
Cell Growth (Normalized to day 0)
Days of treatment (d)
p<0.0001
J
NCI-H211 (SCLC-P)
NCI-H1048 (SCLC-P)
D4
D9
D14
sgNEG
sgCDK1
sgPOU2F3
sgPOU2AF2
sgPOU2AF3
GFP (normalized to day 4)
Empty
HA-dTAG-POU2F3
dTAG-POU2AF2_HA
dTAG-POU2AF3_HA
K
AU-15330 (1μM)
Time (h)
SMARCA4
PBRM1
POU2F3
α–HA
POU2AF2
Vinculin
NCI-H1048 HA dTAG POU2F3
NCI-H1048 HA dTAG POU2AF3
NCI-H526 HA dTAG POU2F3
NCI-H526 HA dTAG POU2AF2
L
NCI-H1048 (SCLC-P) ChIP-seq
POU2F3
POU2F3 (HA-tag)
POU2AF3 (HA-tag)
DMSO
AU-15330
Compacted sites (n = 37063)
-2.5kb
2.5kb
M
POU2F3 target genes loci (NCI-H1048)
5kb
ATAC-seq
POU2F3 ChIP-seq
POU2AF3 ChIP-seq
DMSO
AU-15330
ASCL2
AVIL
GFI1B

Figure S2. Characterization of chromatin accessibility upon mSWI/SNF ATPase degrader treatment and verification of SCLC POU2F3 and POU2AF2/3 dTAG cell lines. Related to Figure 2.

(A) Genome-wide changes in chromatin accessibility upon AU-15330 treatment for four hours in NCI-H526 (SCLC-P) cells along with genomic annotation of sites that lose physical accessibility (lost) or remain unaltered (retained).

(B) Top five *de novo* motifs (ranked by *p-value*) of mSWI/SNF-dependent sites (top) and mSWI/SNF-independent sites (bottom) enriched within AU-15330-loss genomic sites (HOMER, hypergeometric test) in NCI-H526 (SCLC-P) cells. POU2F3 (also known as Oct11) is the top motif.

(C) Genome-wide changes in chromatin accessibility upon AU-15330 treatment for 4 hrs in NCI-H1048 (SCLC-P) cells along with genomic annotation of sites that lose physical accessibility (lost) or remain unaltered (retained).

(D) Top five *de novo* motifs (ranked by *p-value*) of mSWI/SNF-dependent sites (top) and mSWI/SNF-independent sites (bottom) enriched within AU-15330-loss genomic sites (HOMER, hypergeometric test) in NCI-H1048 (SCLC-P) cells. POU2F3 (also known as Oct11) is the top motif.

(E) Genome-wide changes in chromatin accessibility upon AU-15330 treatment for 4 hrs in NCI-H69 (SCLC-A) cells along with genomic annotation of sites that lose physical accessibility (lost) or remain unaltered (retained).

(F) Top five *de novo* motifs (ranked by *p-value*) of mSWI/SNF-dependent sites (top) and mSWI/SNF-independent sites (bottom) enriched within AU-15330-loss genomic sites (HOMER, hypergeometric test) in NCI-H69 (SCLC-A) cells.

(G&H) Immunoblots of POU2F3 and HA-tag validate the expression of exogenous fusion dTAG HA-tag POU2F3 and dTAG HA-tag POU2AF2/3 and the knockout efficacy of endogenous POU2F3 and POU2AF2/3 in NCI-H526 (G) and NCI-H1048 (H) dTAG cells. Vinculin is used as a loading control. This experiment was repeated independently twice.

(I) Relative viability plots after dTAG13 treatment with different time durations. Statistical Analysis was performed using a two-way ANOVA. Data are presented as mean +/- SD (n = 6).

(J) Competition-based proliferation assays in NCI-H211 (left) and NCI-H1048 (right) cells transduced with the indicated sgRNAs with a GFP reporter. The percentage of GFP+ cells correspond to the sgRNA representation within the population. GFP measurements in human cell lines were taken on day 4 post-infection and every four days with Guava Easycyte HT instrument (Millipore). The fold change in GFP+ population (normalized to day 4) was used for analysis. Data are presented as mean +/- SD.

(K) Immunoblots of SMARCA4, PBRM1, POU2F3 and HA-tag validate the regulation of exogenous fusion dTAG HA-tag POU2F3 and dTAG HA-tag POU2AF2/3 in NCI-H526 and NCI-H1048 dTAG cells. Vinculin is used as a loading control.

(L) ChIP–seq read-density heatmaps representing POU2F3 (green), HA-POU2F3 (red), and HA-POU2AF3 (blue) at AU-15330-loss genomic sites in NCI-H1048 cells following treatment with DMSO or 1 μM AU-15330 for 6 hrs.

(M) ATAC-seq and ChIP-seq tracks for *AVIL*, *GFI1B*, and *ASCL2* in NCI-H1048 with and without 1 μM AU-15330 treatment.

Figure S3

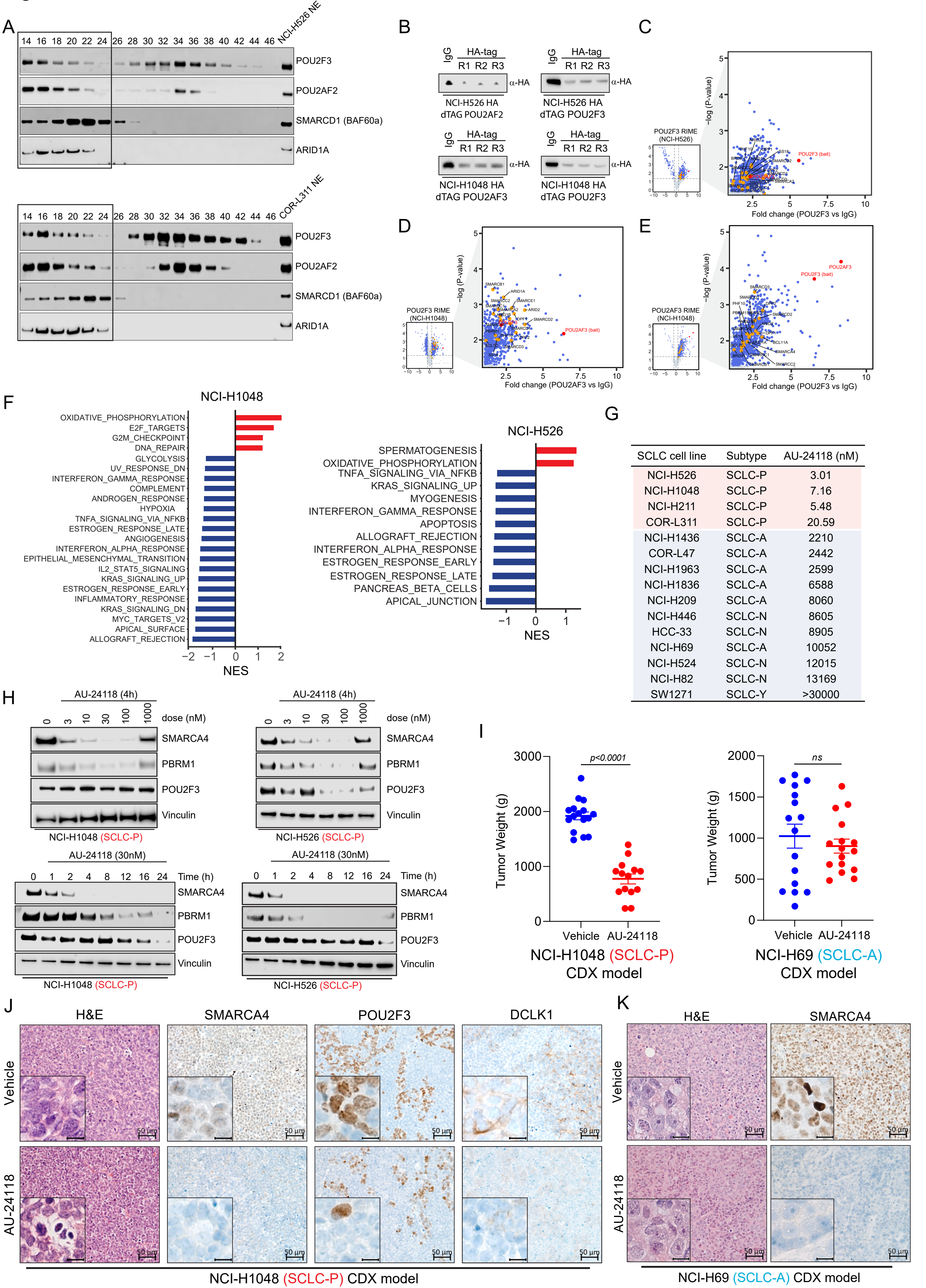

SCLC cell line	Subtype	AU-24118 (nM)
NCI-H526	SCLC-P	3.01
NCI-H1048	SCLC-P	7.16
NCI-H211	SCLC-P	5.48
COR-L311	SCLC-P	20.59
NCI-H1436	SCLC-A	2210
COR-L47	SCLC-A	2442
NCI-H1963	SCLC-A	2599
NCI-H1836	SCLC-A	6588
NCI-H209	SCLC-A	8060
NCI-H446	SCLC-N	8605
HCC-33	SCLC-N	8905
NCI-H69	SCLC-A	10052
NCI-H524	SCLC-N	12015
NCI-H82	SCLC-N	13169
SW1271	SCLC-Y	>30000

Figure S3. Physical interaction of the POU2F3 and mSWI/SNF complexes in SCLC-P cells and selective growth inhibition of AU-24118 in SCLC-P preclinical models. Related to Figures 2-3 and Table S1.

(A) FPLC analysis performed on nuclear extracts of NCI-H526 (SCLC-P) and COR-L311(SCLC-P) cells.

(B) Immunoblot confirming the efficiency of the HA-tag pulldown for HA-POU2F3 and HA-POU2AF2/3 by assessing the presence of residual proteins in the flowthrough samples.

(C-E) TMT-based MS validates the interaction between the mSWI/SNF complex components, POU2F3 and POU2AF2/3 in NCI-H526 (C using HA-POU2F3 as bait), NCI-H1048 (D using HA-POU2AF3 as bait), and NCI-H1048 (E using HA-POU2F3 as bait) (n = 3 biological replicates).

(F) GSEA analysis of 24 hrs of 1 μM AU-15330-induced transcriptomic changes in NCI-H526 and NCI-H1048 cells.

(G) IC_{50} of AU-24118 in a panel of SCLC cell lines after 5 days of treatment.

(H) Immunoblot analysis of indicated proteins in SCLC cells post-treatment with varying time points (bottom) or concentrations (top, four hours) of AU-24118. Vinculin serves as the control for protein loading in all immunoblots.

(I) Individual tumor weight from vehicle and AU-24118 displayed for NCI-H1048 (left) and NCI-H69 (right). Data are presented as mean +/- SEM. T-tests were used to calculate the significance.

(J) Representative H&E staining with corresponding IHC analyses for SMARCA4, POU2F3, and DCLK1 after 5 days of treatment with AU-24118 in NCI-H1048 xenografts (scale=50 μm). The inset scale=20 μm.

(K) Representative H&E staining with corresponding IHC analyses for SMARCA4 after 5 days of treatment with AU-24118 in NCI-H69 xenografts (scale=50 μm). The inset scale=20 μm.

Figure S4

A

SCLC-P cell line	Cisplatin (nM)	Etoposide (nM)
NCI-H526	114.8	3402
NCI-H1048	471.2	297.2
COR-311	528.4	1736
NCI-H211	531.1	1067

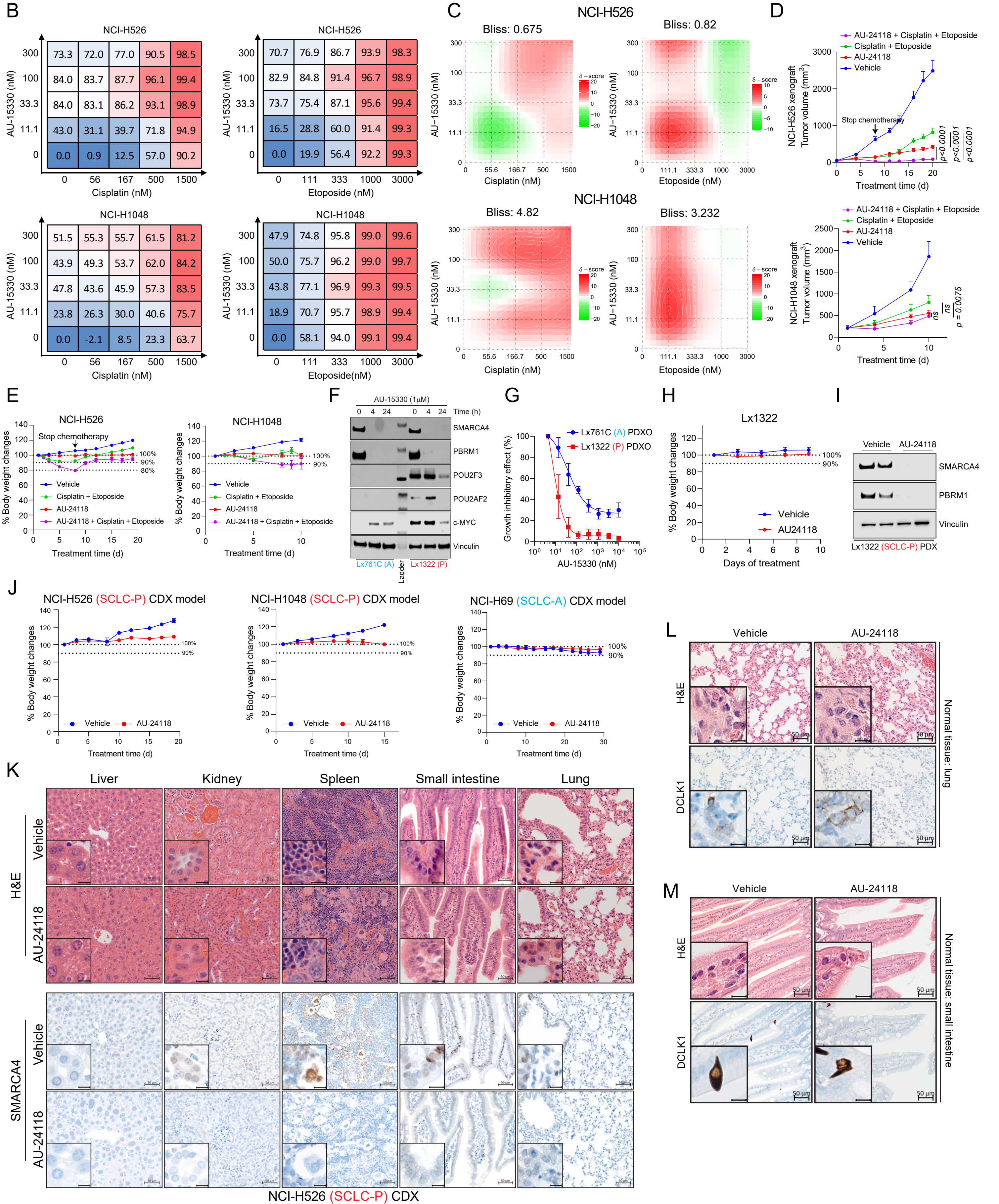

Figure S4. Toxicity and efficacy evaluations of targeting mSWI/SNF complex in SCLC-P preclinical models. Related to Figure 3.

(A) IC_{50} of cisplatin and etoposide (chemotherapy) in a panel of SCLC-P cell lines after 5 days of treatment.

(B) SCLC-P cells were treated with AU-15330 and /or cisplatin and/or etoposide at varied concentrations to determine the inhibition effect.

(C) SCLC-P cells were treated with AU-15330 and /or cisplatin and/or etoposide at varied concentrations to determine drug synergism. Bliss Independent method was used for assessments.

(D) Analysis of tumor volume in indicated SCLC-P xenograft models upon treatment with AU-24118 and/or cisplatin plus etoposide, measured bi-weekly using calipers. Statistical Analysis was performed using a two-way ANOVA. Data are presented as mean +/- SEM.

(E) Percent body weight measurement showing the effect of vehicle, AU-24118, cisplatin plus etoposide and AU-24118 combined with cisplatin plus etoposide throughout the treatment period for indicated SCLC-P xenografts. Data are presented as mean +/- SEM.

(F) Immunoblot analysis of indicated proteins in SCLC cells post-treatment with varying time points of AU-15330 in Lx1322 and Lx761C patient-derived xenograft organoid (PDXO). Vinculin serves as the control for protein loading in all immunoblots.

(G) Lx1322 and Lx761C PDXO showing dose-response curves of AU-15330 at varying concentrations for five days. Data are presented as mean +/- SD (n = 6).

(H) Percent body weight measurement showing the effect of vehicle and AU-24118 throughout the treatment period for Lx1322 xenografts. Data are presented as mean +/- SEM.

(I) Immunoblot illustrating levels of the indicated proteins in Lx1322 after 5 days of AU-24118 administration. Vinculin is utilized as the loading control across immunoblots. PDX, patient- derived xenograft.

(J) Percent body weight measurement showing the effect of vehicle and AU-24118 throughout the treatment period for NCI-H526 (left panel), NCI-H1048 (middle panel), and NCI-H69 (right panel) xenografts. Data are presented as mean +/- SEM.

(K) Representative H&E staining with corresponding IHC analyses for SMARCA4 in normal organs (liver, spleen, kidney, small intestine, and lung) (scale=50 μm). The inset scale=20 μm.

(L) Representative H&E staining of murine lung with corresponding tuft cell marker DCLK1 IHC after *in vivo* administration of AU-24118 at study endpoint (scale=50 μm). Magnified views of lung alveolar epithelium in H&E and corresponding DCLK1 IHC shown in insets (scale=20 μm).

(M) Representative H&E staining of murine small intestine with corresponding tuft cell marker DCLK1 IHC after *in vivo* administration of AU-24118 at study endpoint (scale=50 μm). Magnified views of intestinal enterocytes in H&E and corresponding DCLK1 IHC shown in insets (scale=20 μm).

Figure S5

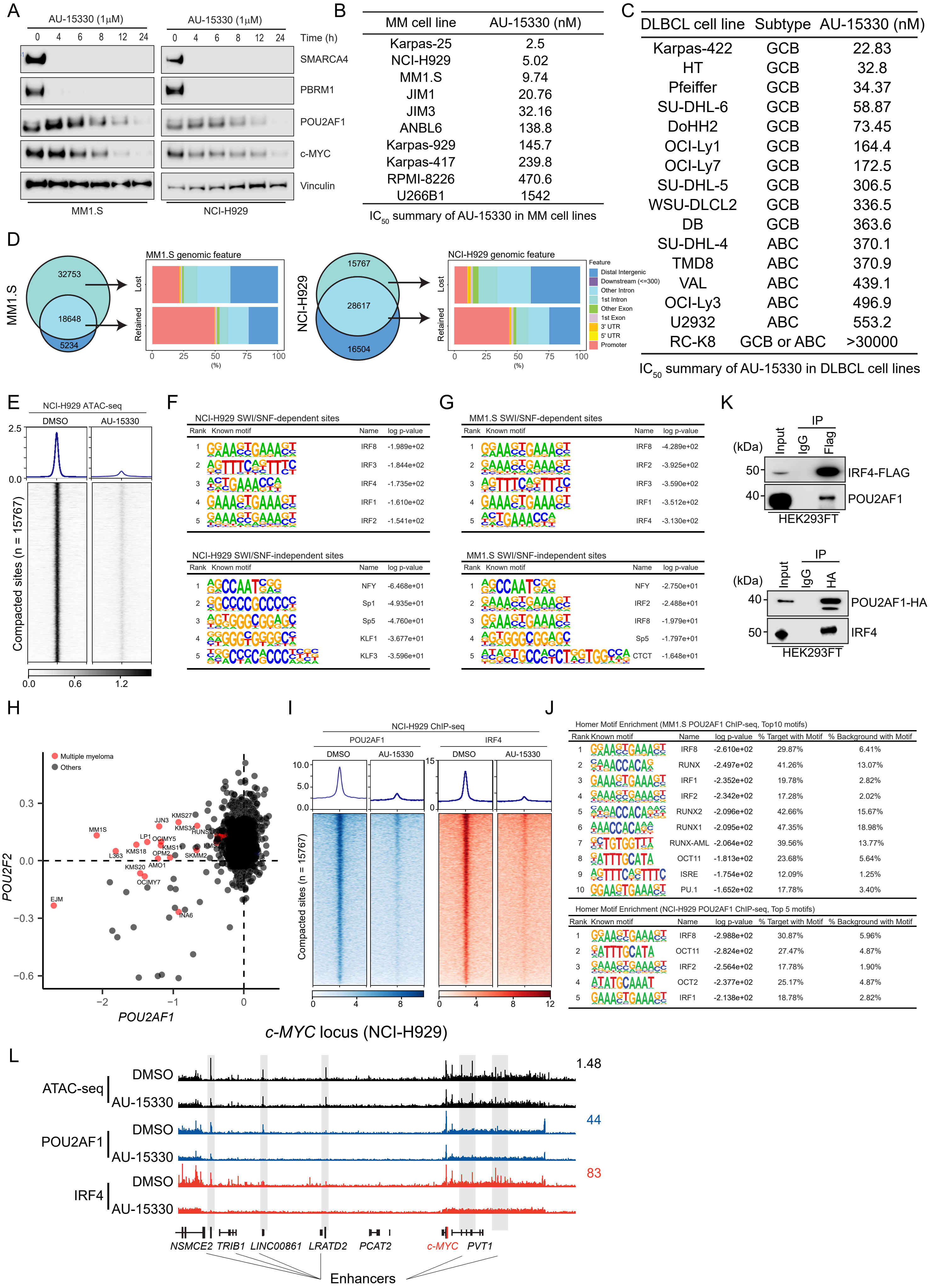
A
AU-15330 (1μM)
AU-15330 (1μM)
0 4 6 8 12 24
Time (h)
SMARCA4
PBRM1
POU2AF1
c-MYC
Vinculin
MM1.S
NCI-H929
B
MM cell line
AU-15330 (nM)
Karpas-25 2.5
NCI-H929 5.02
MM1.S 9.74
JIM1 20.76
JIM3 32.16
ANBL6 138.8
Karpas-929 145.7
Karpas-417 239.8
RPMI-8226 470.6
U266B1 1542
IC50 summary of AU-15330 in MM cell lines
C
DLBCL cell line
Subtype
AU-15330 (nM)
Karpas-422 GCB 22.83
HT GCB 32.8
Pfeiffer GCB 34.37
SU-DHL-6 GCB 58.87
DoHH2 GCB 73.45
OCI-Ly1 GCB 164.4
OCI-Ly7 GCB 172.5
SU-DHL-5 GCB 306.5
WSU-DLCL2 GCB 336.5
DB GCB 363.6
SU-DHL-4 ABC 370.1
TMD8 ABC 370.9
VAL ABC 439.1
OCI-Ly3 ABC 496.9
U2932 ABC 553.2
RC-K8 GCB or ABC >30000
IC50 summary of AU-15330 in DLBCL cell lines
D
MM1.S
32753
18648
5234
MM1.S genomic feature
Lost
Retained
NCI-H929
15767
28617
16504
NCI-H929 genomic feature
Feature
Distal Intergenic
Downstream (<=300)
Other Intron
1st Intron
Other Exon
1st Exon
3' UTR
5' UTR
Promoter
E
NCI-H929 ATAC-seq
DMSO
AU-15330
Compacted sites (n = 15767)
F
NCI-H929 SWI/SNF-dependent sites
NCI-H929 SWI/SNF-independent sites
G
MM1.S SWI/SNF-dependent sites
MM1.S SWI/SNF-independent sites
K
(kDa)
Input
IP
IgG
Flag
IRF4-FLAG
POU2AF1
HEK293FT
HA
POU2AF1-HA
IRF4
H
Multiple myeloma
Others
POU2F2
POU2AF1
I
NCI-H929 ChIP-seq
POU2AF1
IRF4
DMSO
AU-15330
Compacted sites (n = 15767)
J
Homer Motif Enrichment (MM1.S POU2AF1 ChIP-seq, Top10 motifs)
Homer Motif Enrichment (NCI-H929 POU2AF1 ChIP-seq, Top 5 motifs)
c-MYC locus (NCI-H929)
L
ATAC-seq
DMSO
AU-15330
POU2AF1
IRF4
1.48
44
83
NSMCE2
TRIB1
LINC00861
LRATD2
PCAT2
c-MYC
PVT1
Enhancers

Figure S5. Selective essentiality of the mSWI/SNF complex in B cell malignancies and epigenetic landscape changes in multiple myeloma cells upon treatment with the mSWI/SNF ATPase degrader. Related to Figure 4.

(A) Immunoblot analysis of indicated proteins in MM1.S and NCI-H929 cells post-treatment with varying time points of 1μM AU-15330. Vinculin serves as the control for protein loading in all immunoblots.

(B) IC_{50} of AU-15330 in a panel of multiple myeloma cell lines after 5 days of treatment.

(C) IC_{50} of AU-15330 in a panel of diffuse large B cell lymphoma (DLBCL) cell lines after 5 days of treatment. ABC, activated B cell-like subtype lymphoma; GCB, germinal center B-cell-like subtype lymphoma.

(D) Genome-wide changes in chromatin accessibility upon AU-15330 treatment for 4 hours in MM1.S (left) and NCI-H929 (right) cells along with genomic annotation of sites that lose physical accessibility (lost) or remain unaltered (retained).

(E) Reduced chromatin accessibility induced by mSWI/SNF ATPase degradation. Visualization of ATAC-seq read-density in NCI-H929 cells post-treatment for 4 hours with either vehicle or 1μM AU-15330 (n = 2 biological replicates).

(F&G) Top five *de novo* motifs (ranked by *p-value*) enriched within AU-15330-loss genomic sites (HOMER, hypergeometric test) in NCI-H929 (F) and MM1.S cells (G).

(H) Scatter plot showing the dependency scores for POU2F2/POU2AF1 in multiple myeloma (red) and other cancer types based on DepMap dataset.

(I) ChIP–seq read-density heatmaps representing POU2AF1 (blue) and IRF4 (red) at AU-15330-loss genomic sites in NCI-H929 cells following 6 hrs treatment with DMSO or 1 μM AU-15330.

(J) Top *de novo* motifs (ranked by p-value) enriched within POU2AF1 binding sites (HOMER, hypergeometric test) in MM1.S cells (upper) and NCI-H929 cells (bottom).

(K) Co-immunoprecipitation (IP) of tagged POU2AF1 and IRF4 followed by immunoblot for indicated proteins in HEK293FT cells. This experiment was repeated independently twice.

(L) Combined ATAC-seq and ChIP-seq tracks for *c-MYC* locus in NCI-H929 cells with and without AU-15330 treatment.

Figure S6

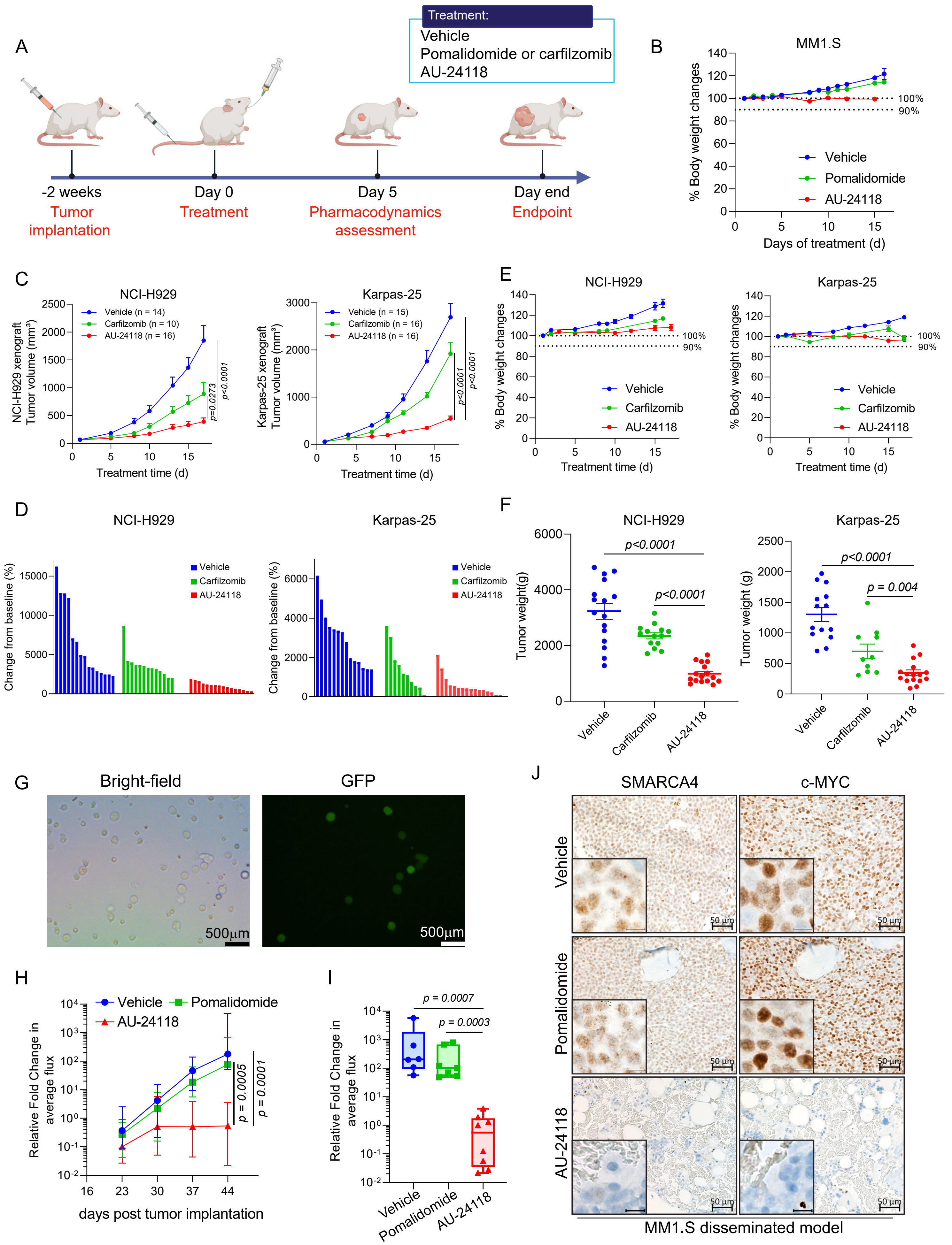
A
Treatment:
Vehicle
Pomalidomide or carfilzomib
AU-24118
-2 weeks
Tumor implantation
Day 0
Treatment
Day 5
Pharmacodynamics assessment
Day end
Endpoint
B
MM1.S
% Body weight changes
Days of treatment (d)
Vehicle
Pomalidomide
AU-24118
100%
90%
C
NCI-H929
NCI-H929 xenograft Tumor volume (mm³)
Vehicle (n = 14)
Carfilzomib (n = 10)
AU-24118 (n = 16)
p=0.0273
p<0.0001
Treatment time (d)
Karpas-25
Karpas-25 xenograft Tumor volume (mm³)
Vehicle (n = 15)
Carfilzomib (n = 16)
AU-24118 (n = 16)
p<0.0001
p<0.0001
Treatment time (d)
E
NCI-H929
Karpas-25
% Body weight changes
Vehicle
Carfilzomib
AU-24118
Treatment time (d)
D
NCI-H929
Karpas-25
Change from baseline (%)
Vehicle
Carfilzomib
AU-24118
F
NCI-H929
Karpas-25
Tumor weight(g)
Tumor weight (g)
p<0.0001
p<0.0001
p = 0.004
Vehicle
Carfilzomib
AU-24118
G
Bright-field
GFP
500µm
500µm
H
Vehicle
Pomalidomide
AU-24118
Relative Fold Change in average flux
p = 0.0005
p = 0.0001
days post tumor implantation
I
p = 0.0007
p = 0.0003
Relative Fold Change in average flux
Vehicle
Pomalidomide
AU-24118
J
SMARCA4
c-MYC
Vehicle
Pomalidomide
AU-24118
50 µm
MM1.S disseminated model

Figure S6. Efficacy of AU-24118 in multiple myeloma subcutaneous xenografts and disseminated tumor model. Related to Figure 5.

(A) Overview of the AU-24118 efficacy study conducted using multiple myeloma subcutaneous xenograft models.

(B) Percent body weight measurement showing the effect of vehicle, carfilzomib, pomalidomide, or AU-24118 throughout the treatment period in MM1.S xenograft models. Data are presented as mean+/- SEM.

(C) Analysis of tumor volumes in indicated multiple myeloma xenograft models upon treatment with AU-24118 or carfilzomib, measured bi-weekly using calipers in the NCI-H929 and Karpas-25 xenografts. Statistical Analysis was performed using a two-way ANOVA. Data are presented as mean+/- SEM.

(D) Waterfall plots depicting change in tumor volume at the study endpoint for NCI-H929 and Karpas-25-derived xenograft models.

(E) Percent body weight measurement showing the effect of vehicle, carfilzomib, pomalidomide, or AU-24118 throughout the treatment period in NCI-H929, Karpas-25, and MM1.S xenograft models. Data are presented as mean +/- SEM.

(F) Individual tumor weights from vehicle, carfilzomib, and AU-24118 treated mice from the NCI-H929 and Karpas-25 xenograft study. Data are presented as mean +/- SEM. T-tests were used to calculate the significance.

(G) Representative images of GFP/luciferase-expressing MM1.S cells extracted from femur to verify the MM1.S disseminated model.

(H) Quantification of bioluminescence signal indicating the tumor burden (measured once per week using the IVIS Spectrum In Vivo Imaging System) in the MM1.S disseminated xenograft model under different treatments. Statistical Analysis was performed using a two-way ANOVA. Data are presented as mean +/- SEM.

(I) Box plot of the bioluminescence quantification at endpoint (day 44) in the MM1.S disseminated xenograft model. T-tests were used to calculate the significance. The whiskers extend from the minimum to the maximum values, indicating the full range of the data. The middle line represents the median of the data. The box spans from the first quartile (Q1, 25th percentile) to the third quartile (Q3, 75th percentile), representing the interquartile range (IQR).

(J) Representative IHC analyses for SMARCA4 and c-Myc after 5 days of the indicated treatment in MM1.S disseminated xenografts (scale=50 μm). The inset scale=20 μm.

Figure S7

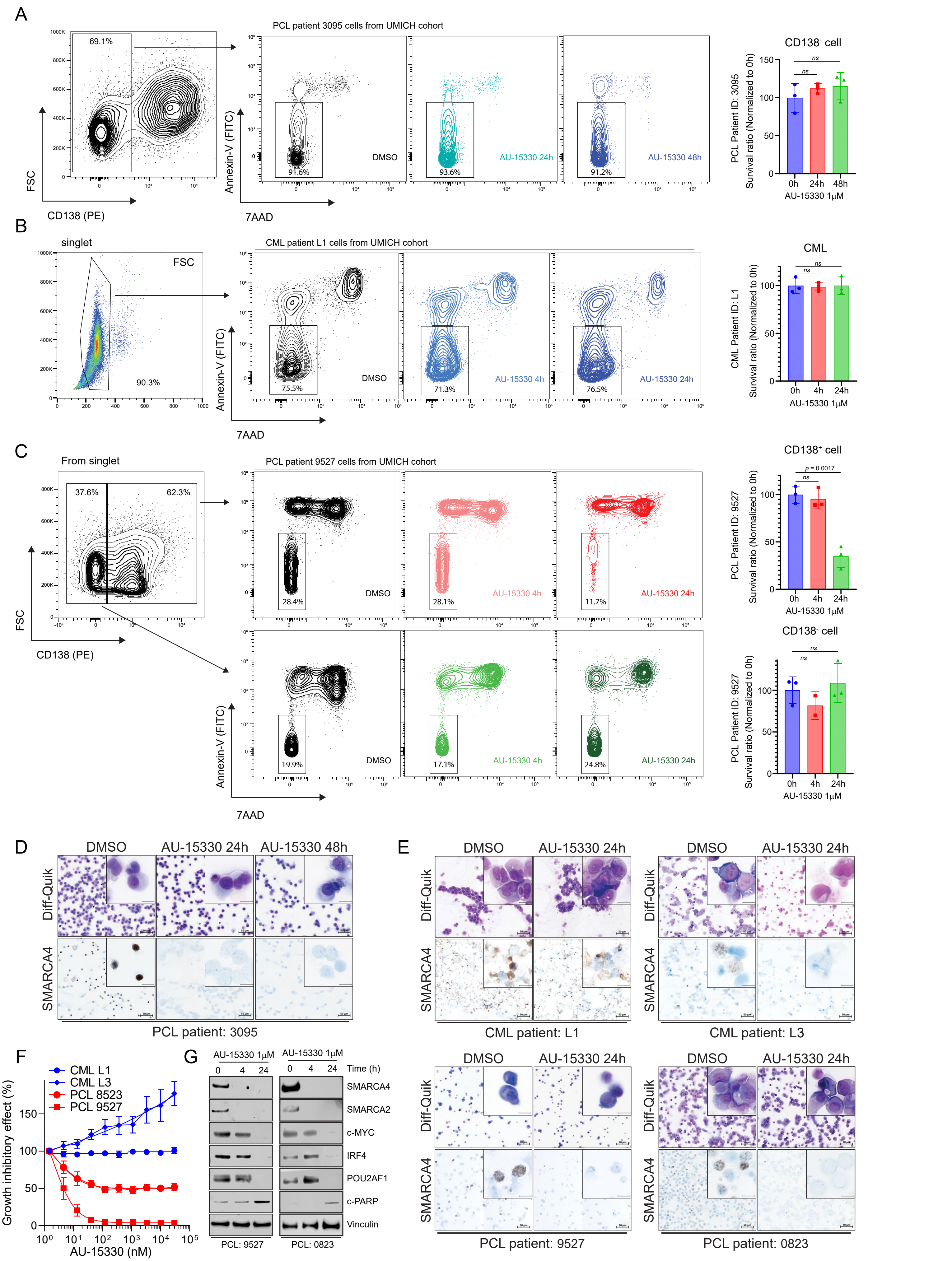

A
PCL patient 3095 cells from UMICH cohort
69.1%
FSC
CD138 (PE)
Annexin-V (FITC)
7AAD
91.6%
DMSO
93.6%
AU-15330 24h
91.2%
AU-15330 48h
CD138- cell
PCL Patient ID: 3095
Survival ratio (Normalized to 0h)
ns
0h
24h
48h
AU-15330 1μM
B
singlet
FSC
90.3%
CML patient L1 cells from UMICH cohort
75.5%
DMSO
71.3%
AU-15330 4h
76.5%
AU-15330 24h
CML
CML Patient ID: L1
C
From singlet
37.6%
62.3%
PCL patient 9527 cells from UMICH cohort
28.4%
28.1%
11.7%
19.9%
17.1%
24.8%
CD138+ cell
p = 0.0017
PCL Patient ID: 9527
CD138- cell
D
DMSO
AU-15330 24h
AU-15330 48h
Diff-Quik
SMARCA4
PCL patient: 3095
E
CML patient: L1
CML patient: L3
PCL patient: 9527
PCL patient: 0823
F
CML L1
CML L3
PCL 8523
PCL 9527
Growth inhibitory effect (%)
AU-15330 (nM)
G
AU-15330 1μM
Time (h)
SMARCA4
SMARCA2
c-MYC
IRF4
POU2AF1
c-PARP
Vinculin
PCL: 9527
PCL: 0823

Figure S7. Targeting the mSWI/SNF complex in cells from multiple myeloma and chronic myelogenous leukemia (CML) patients. Related to Figure 5 and Table S2.

(A) Representative images (left) and quantification (right) of flow cytometry measuring the proportion of Annexin-V (+) and/or 7AAD (+) cells in CD138 (-) cells from plasma cell leukemia (PCL) patient PCL3095 treated with AU-15330 for the indicated time. Data are presented as mean +/- SD (n = 3). T-tests were used to calculate the significance. The same patient (3095) bulk cell population data was used in Figure 5J.

(B) Representative images (left) and quantification (right) of flow cytometry measuring the proportion of Annexin-V (-) and/or 7AAD (-) cells from CML L1 patient treated with AU-15330 for the indicated time. T-tests were used to calculate the significance. Data are presented as mean +/- SD (n = 3).

(C) Representative images (left) and quantification (right) of flow cytometry measuring the proportion of Annexin-V (-) and/or 7AAD (-) cells in CD138 (-) or CD138 (+) cells from PCL 9527 patient treated with AU-15330 for the indicated time. T-tests were used to calculate the significance. Data are presented as mean +/- SD (n = 3).

(D) Representative Diff-Quik staining with corresponding ICC images demonstrating abrogation of expression of SMARCA4 after 24h or 48h of treatment with AU-15330 in PCL 3095 fresh patients cells in comparison to DMSO (scale=50 μm). The inset scale=20 μm.

(E) Representative Diff-Quik staining with corresponding ICC analyses for SMARCA4 after 4h or 24h of treatment with AU-15330 in two individual CML (top) and two individual PCL (bottom) patients' frozen cells (scale=50 μm). The inset scale=20 μm.

(F) Two individual CML and two individual PCL patients' frozen cells showing dose-response curves of AU-15330 at varying concentrations for two days. Data are presented as mean +/- SD (n = 6).

(G) Immunoblot illustrating levels of the indicated proteins in PCL patients' cells after AU-15330 treatment for 4h or 24h. Vinculin is utilized as the loading control.

Table S2. Detailed patient information. Related to STAR Methods.

Sample ID	Collection date	Samples status	Sex	Age of patient	Sample type	% Plasma cells	Disease	Kappa/ lambda	Cytogenetics	FISH	Treatment	Status
9527	3/31/2016	Frozen	F	72	PB	84	PCL	kappa	ND	ND	Revlimid Velcade Dex	Relapsed
0823	5/20/2013	Frozen	M	68	PB	78	PCL	kappa	46,XY[19]/78,XXY,+X,+1,-2,-3,+4,+4,-5,-5,+7,+7,+7,-9,+10,+10,-11,+15,+17,-18,+19,+19,+20,+22,+22[1]	t(4;14)	NA	Naïve
3095	4/2/2024	Fresh	F	75	PB	23	PCL	kappa	NA	Evidence for a gain of 1q (CKS1B), the IGH::FGFR3 gene fusion, and monosomy 13	Revlimid Velcade Dex	Relapsed
CML-L1	4/13/2009	Frozen	NA	NA	NA	NA	CML	NA	NA	BCR/ABL gene fusion	NA	NA
CML-L3	4/18/2008	Frozen	NA	NA	NA	NA	CML	NA	NA	BCR/ABL gene fusion	NA	NA

Table S3. Detailed antibody information. Related to STAR Methods.

Antigen	Vendor	Catalog number	RRID	Application	Notes
SMARCA2/BRM	Bethyl Laboratories	A301-016A	AB_2193933	Western Blot	1:1000
SMARCA4/BRG1	Cell Signaling Technology	52251S	AB_2799410	Western Blot	1:1000
PBRM1	Bethyl Laboratories	A301-591A	AB_1078808	Western Blot	1:1000
Vinculin	Cell Signaling Technology	18799S	AB_2714181	Western Blot	1:1000
c-MYC	Abcam	ab32072	AB_731658	Western Blot	1:1000
Cleaved PARP (Asp214)	Cell Signaling Technology	9541	AB_331426	Western Blot	1:1000
POU2F3	Cell Signaling Technology	92579		Western Blot	1:1000
ASCL1	Abcam	ab74065	AB_1859937	Western Blot	1:1000
POU2AF2	Cell Signaling Technology	20217s		Western Blot	1:1000
N-MYC	Santa Cruz	sc-53993	AB_831602	Western Blot	1:1000
GFI1B	Santa Cruz	sc-28356	AB_2110132	Western Blot	1:1000
NEUROD1	Abcam	ab60704	AB_943491	Western Blot	1:1000
HA-tag	Cell Signaling Technology	3724s	AB_1549585	Western Blot, ChIP, RIME	WB: 1:1000, ChIP: 4ug/2M cells, RIME: 20ug/reaction
SMARCD1	Santa Cruz	sc-135843	AB_2192137	Western Blot	1:1000
POU2AF1	Cell Signaling Technology	43079s		Western Blot	1:1000
IRF4	Cell Signaling Technology	4964s	AB_10698467	Western Blot, ChIP	WB: 1:1000, ChIP: 4ug/2M cells
ARID1A	Santa Cruz	sc-373784	AB_10917727	Western Blot	1:1000
DCLK1	Abcam	ab109029	AB_10864128	IHC	
BRG1	Abcam	ab108318	AB_10889900	IHC	
POU2F3	Cell Signaling Technology	mAB#36135/clone E5N2D	AB_2924784	IHC, ChIP	ChIP: 4ug/2M cells
CD38	Ventana	760-4785/clone SP149		IHC	
POU2AF1	Thermo Fisher Scientific	PA5-121026	AB_2914598	ChIP, RIME	ChIP: 4ug/2M cells, RIME: 20ug/reaction
CD138	Miltenyi Biotec	130-119-840	AB_2751879	Flow	2uL/reaction
